# Recent Advances in Functionalized Biomass‐Derived Porous Carbons and their Composites for Hybrid Ion Capacitors

**DOI:** 10.1002/advs.202406235

**Published:** 2024-07-19

**Authors:** Nithya S. George, Gurwinder Singh, Rohan Bahadur, Prashant Kumar, Kavitha Ramadass, CI Sathish, Mercy Benzigar, Davidson Sajan, Arun Aravind, Ajayan Vinu

**Affiliations:** ^1^ Global Innovative Centre for Advanced Nanomaterials (GICAN) College of Engineering, Science and Environment (CESE) School of Engineering The University of Newcastle Callaghan NSW 2308 Australia; ^2^ Centre for Advanced Functional Materials Department of Physics Bishop Moore College Mavelikara Alappuzha Kerala 690110 India

**Keywords:** biomass‐derived porous carbons, electrochemistry, monovalent HICs, multivalent HICs, surface functionalization

## Abstract

Hybrid ion capacitors (HICs) have aroused extreme interest due to their combined characteristics of energy and power densities. The performance of HICs lies hidden in the electrode materials used for the construction of battery and supercapacitor components. The hunt is always on to locate the best material in terms of cost‐effectiveness and overall optimized performance characteristics. Functionalized biomass‐derived porous carbons (FBPCs) possess exquisite features including easy synthesis, wide availability, high surface area, large pore volume, tunable pore size, surface functional groups, a wide range of morphologies, and high thermal and chemical stability. FBPCs have found immense use as cathode, anode and dual electrode materials for HICs in the recent literature. The current review is designed around two main concepts which include the synthesis and properties of FBPCs followed by their utilization in various types of HICs. Among monovalent HICs, lithium, sodium, and potassium, are given comprehensive attention, whereas zinc is the only multivalent HIC that is focused upon due to corresponding literature availability. Special attention is also provided to the critical factors that govern the performance of HICs. The review concludes by providing feasible directions for future research in various aspects of FBPCs and their utilization in HICs.

## Graphical abstract

This review covers the synthesis and properties of functionalized biomass‐derived porous carbons, and their application as cathodic, anodic and/or dual electrode material for Lithium, Sodium and Potassium based monovalent hybrid ion capacitors and Zinc‐based multivalent hybrid ion capacitors.

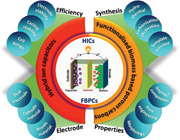

## 1 Introduction

The increasing energy demands, escalating fossil fuel usage and environmental challenges have created the need for the development of sustainable energy solutions.^[^ ^1^ ^]^ One of the critical aspects in this space is to develop state‐of‐the‐art devices that can store and deliver energy and power efficiently and among such devices, batteries and supercapacitors are the leading contenders. Abundant research is being pursued in this field with a major focus on improving the power density and energy density in batteries and supercapacitors respectively.^[^ ^2^ ^]^ The development of effective energy storage systems that can utilize both batteries and supercapacitors in a single system is gaining popularity as a hot research topic.^[^ ^3^ ^]^ Such a system, called a hybrid ion capacitor (HIC), can significantly boost the energy density and power density along with cyclic stability.^[^ ^4^ ^]^ Therefore, substantial research has been devoted in this field to investigate either monovalent metal ion HICs utilizing alkali metal ions (Li^+^, Na^+^, K^+^) due to their exceptional energy storage capabilities^[^ ^5^ ^]^ or multivalent metal ion HICs, (Ca^2+^, Mg^2+^, Zn^2+^ and Al^3+^)_._^[^ ^6^, ^7^ ^]^

Electrode materials play a crucial role in influencing the overall electrochemical performance of any energy storage device, be it batteries,^[^ ^8^ ^]^ supercapacitors^[^ ^9^ ^]^ or HICs.^[^ ^10^ ^]^ In a typical HIC, a battery‐type electrode (negative electrode/anode), and a capacitor‐type electrode (positive electrode/cathode) are placed in a solution of metal ion‐based electrolyte.^[^ ^11^ ^]^ In recent times, a variety of anode materials such as carbon‐based materials, metal oxides, sulfides, MXenes and metal phosphides have been utilized in HICs.^[^ ^12^ ^]^ The use of pure metals as anode materials, for example, Zn and, is a feasible operation for multivalent HICs, however, employing monovalent metals for anode electrode fabrication is impractical due to their high reactivity.^[^ ^13^ ^]^ This necessitates the use of materials such as activated carbon, metal oxides and MXene‐based electrodes in monovalent HICs.

Carbon‐based materials have garnered significant attention and their extensive exploration as electrode materials is largely credited to their unique properties such as tunable porosity, ease of surface chemistry manipulation, and high thermal and chemical stability.^[^ ^14^ ^]^ The Pore architecture of porous carbons is a crucial factor affecting electrochemical behavior. For example, smaller pores control ion diffusion through ion dissolution, while larger pores promote the diffusion of larger solvated ions^[^ ^8a^ ^]^ and both these qualities improve the overall electrochemical performance of any energy storage device. As an illustration, micro and mesoporous carbons derived from kapok fruit peel exhibited a high specific capacitance of 332.3 F g^−1^ at 1 A g^−1^ with energy and power densities of 12.36 Wh kg^−1^ and 500 W kg^−1^ respectively.^[^ ^15^ ^]^ This biomass presented a honeycomb‐like structure with an interconnected pore network that can be suitably engineered by altering the potassium hydroxide (KOH) impregnation quantities. The type of functional groups anchored on the surface of porous carbon is also a dominant factor that affects the conductivity and overall electrochemical performance.^[^ ^16^ ^]^ For instance, porous carbon derived from egg white showed a high surface area (3898 m^2^ g^−1^) and appreciable N doping, a combination which produced a promising outcome for energy density (124.7 Wh kg^−1^) at a power density 2547 W kg^−1^ as a lithium‐ion capacitor (LIC) device.^[^ ^17^ ^]^ The above findings suggest that it is significant to address the pore structure and surface functionalization in a carbon‐based electrode by innovating new and advanced materials. Typical carbon materials used as electrochemical electrodes include carbon nanotubes,^[^ ^18^ ^]^ graphite^[^ ^19^ ^]^ and different types of nanoporous carbons.^[^ ^20^ ^]^

Among these carbon‐based electrodes, nanoporous carbons have emerged as promising materials for HICs as these are low‐cost, can be sourced from a range of carbonaceous raw materials and be easily converted into porous carbons with desirable physico‐chemical properties. Among various precursors, biomass is an intriguing raw material due to its low cost and abundant availability and the porous carbons derived from these biomass materials as the electrode materials have been reported on numerous occasions.^[^ ^21^ ^]^ High surface area, hierarchical porosity and surface functional groups are typical properties of the functionalized porous carbons developed from biomass (FBPCs).^[^ ^8^, ^22^ ^]^ Surface functionalization with heteroatoms is an exquisite quality in FBPCs and this is largely possible as the biomass can inherently contain heteroatoms such as oxygen and nitrogen which upon doping in porous carbon can create a large number of active sites and promote the electron transfer by enhancing its conductivity, and consequently increase the electrochemical performance.^[^ ^23^ ^]^ Such FBPCs can also offer expanded interlayer spacing as compared to graphite, facilitating ion insertion/extraction, and thus acting as sustainable and green electrodes in electrochemical energy storage (EES) devices. For instance, the N and O co‐doped FBPC‐based sodium ion capacitor (SIC) device delivered a promising energy density of 36k Wh kg^−1^ at a high‐power density of 53k W kg^−1^ which was made possible due to the surface enrichment with the heteroatoms.^[^ ^24^ ^]^ The SIC device also showed good cyclic stability with 90.5% capacitance retention even after 8000 cycles at a high current density of 5 A g^−1^. These FBPCs can also be tailored to vary their morphology, through suitable alterations during the synthesis, which can lead to better accessibility of ions and hence increased electrochemical performance. For instance, an ultra‐thin 2D FBPC developed from agroforestry biomass was doped with N to deliver an impressive surface area (1802 m^2^ g^−1^) and specific capacity of 67 mAh g^−1^ at 0.1 A g^−1^.^[^ ^25^ ^]^ Lignin‐derived porous carbon (LPC) was manipulated for its pore structure via molecular level strategy during the carbonization cum activation process and utilized as a cathode in LIC which delivered a promising energy density of 153.9 Wh kg^−1^ and power density of 22.4k W kg^−1^.^[^ ^26^ ^]^ FBPCs are also a good choice for fabricating hybrid or composite materials wherein the addition of external species brings an increase in electrochemical efficiency. For instance, bamboo‐derived FBPC coupled with nano‐ Si obtained by magnesiothermic reduction reaction for LIC device delivered a high energy density of 244 Wh kg^−1^ along with a power density of 127 W kg^−1^.^[^ ^27^ ^]^ From these examples, it can be confirmed that the fascinating structural and chemical aspects of FBPC allow for them to be used as cathode, anode and dual carbon electrodes in most monovalent HICs and some multivalent HICs.

**Figure 1:**
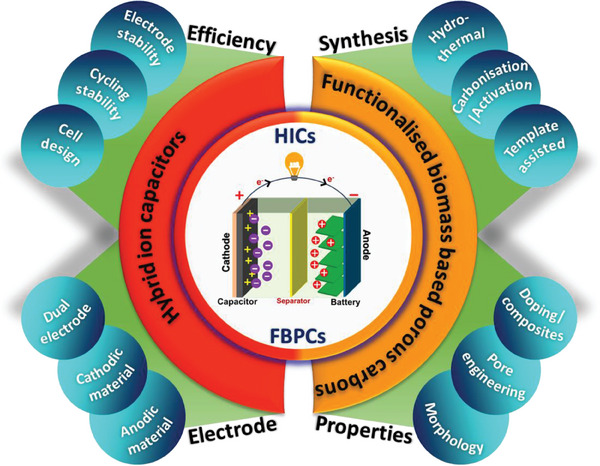
Overview of the review in terms of various attributes of materials and HICs.

This review is dedicated to the discussion on FBPCs including their synthesis and structure design criteria and their application for monovalent HICs including LICs, SICs, potassium ion capacitors (KICs) and multivalent HICs including zin ion capacitors (ZICs). The review contents and the included literature have been carefully chosen to shed light on the recent highlights in the field and not repeat what already exists in the literature. We examined the recent literature and found that related reviews have focused on improving the energy density of the HIC devices as compared to the symmetric carbon‐based supercapacitor electrodes.^[^ ^2^, ^4^, ^5^, ^6^, ^7^, ^14^, ^28^ ^]^ Although researchers have focused on finding a suitable combination of battery‐type and capacitor‐type electrode material to avoid the kinetic mismatch between the electrodes, achieving both high energy density and power density together for the combined system is still challenging. Our review is different from the previous ones as we have focused on how FBPC‐based electrodes can be employed for enhancing the electrochemical performance of various HICs. We also made attempts to classify the FBPCs electrochemical performance based on various electrode configurations including cathode, anode and dual carbon. This review will provide readers with a broad understanding of the various critical factors that control the electrochemical performance of both monovalent HICs and multivalent HICs when the electrode materials are derived from FBPCs. Overall, this review is sufficiently placed in terms of its timely requirement and the covered contents. An overview of the review is presented in **Figure** **1**.

## 2 Strategies to Fabricate FBPCs

FBPCs, in general, are materials with high surface area and different functional moieties including heteroatoms and surface functional organic groups. Like any other carbon‐based material, high temperature is a prerequisite for the conversion of biomass into a porous carbon material which can be further tailored into the desired type of porosity and surface functionalization by employing activation with either physical or chemical agents. This section is devoted to the synthesis methods for FBPCs and will discuss and compare the various methods and obtained materials to provide insights into the current developments in this area. A summary and relative comparison of the characteristics, advantages and disadvantages of the three synthesis methods including hydrothermal carbonization (HTC), carbonization and activation and template‐assisted are also provided in **Table** **1**.

**Table 1:** A comparative analysis of various characteristics, advantages, and disadvantages of the three methods of synthesis of FBPCs.

Characteristics	Advantages	Disadvantages
Hydrothermal carbonization (HTC)		
Mild reaction temperatures (180–220 °C) and high pressures (10–20 bar) are required for the operation	Pre‐drying treatment of biomass is not required which reduces energy consumption, saves time and the cost	Limited surface area and porosity in hydrochar require integration using chemical or physical activation
Suitable for a wide variety of biomass including woody and non‐woody ones and most plant and animal wastes	Overall, low energy consumption as compared to conventional pyrolysis for obtaining a high carbon yield	Expensive high‐pressure autoclaves are required that often need replacement due to wear and tear which increases the cost
Carried out in an aqueous phase as the primary media and other additives such as dilute acidic/alkaline/organic co‐solvents can also be used as per requirement	Efficient technique for hydrolysis, dehydration, decarboxylation, polymerization, condensation and polymerization of biomass to form FBPCs	Scaling up at commercial levels is difficult and poses several challenges including process and product optimization, high capital and operations costs
Formed hydrochar has higher carbon content, good thermal stability, hydrophobicity and higher energy density as compared to source biomass	Suitable for metal/heteroatom loading/achieving specific morphology such as carbon spheres/fibres and reducing ash content and volatiles in FBPCs	Hydrochar, itself has a limited market due to inferior quality for applications and standardization issues which makes the process economically unviable
Hydrochar is an appealing precursor for further modification/functionalization	Non‐toxic, less waste and low byproduct emission and hence is sustainable	Hydrochar properties may not be uniform as they depend on the feedstock biomass
Carbonization and activation		
Carbonization involves pyrolysis of raw biomass to produce biochar at high temperatures under inert conditions	Carbonization is a versatile operation to convert biomass into high‐carbon content in biochar for further modification	High‐temperature requirements can contribute towards high costs for mass‐scale production at commercial levels
Activation can be used either for raw biomass or biochar and can involve chemical or physical activating agents in either solid or liquid state	A combination of carbonization and activation typically yields high surface area and tunable pore characteristics such as pore volume and pore size	The complex/corrosive acidic/alkaline activating agents can cause equipment deterioration and can also lead to the formation of undesirable volatiles
Carbonization condenses/concentrates the carbon in biomass to an increased level whereas activation yields a porous structure within the carbon framework	Activation spans across a wide range of agents including physical (CO_2_ and steam) and chemical (KOH, ZnCl_2_ and H_3_PO_4_) to yield variation in porosity	Biomass is usually wet and will need to be pre‐dried before proceeding with the carbonization cum activation which can increase cost and time
Carbonization and activation can both be controlled via adjustment of the experimental conditions including the amount of activation and temperature	The FBPCs produced carbonization cum activation are thermally and chemically stable and can be utilized for a myriad of application fields	Substantial capital investment is required to set up the facilities for the large‐scale production of FBPCs via carbonization cum activation procedures
Template‐assisted		
Templating is a facile ploy to construct FBPCs with a diverse range of structures, morphologies, and porosity	Templating produced FBPCs with a uniform pore structure which is a crucial requirement in several applications	Harsh acidic or alkaline requirements for the removal of templates was a big environmental concern
Surfactants, block co‐polymers, silica, and metal salts are often employed as templating agents	Templating allows for the creation of FBPCs with good thermal and chemical stability	The structural integrity of FBPCs could be compromised during templating removal and the template itself is hard to recover
Highly ordered materials with well‐defined morphology can be obtained by controlling the growth parameters	Templating allows for the creation of surface functional groups with ease and in the desired concentration in FBPCs	Templating can require several steps and the use of high temperatures which makes the process time‐consuming
Soft and hard templating allows the manipulation of various physico‐chemical properties of FBPCs	The size and type of the template can be varied to produce FBPCs with desired surface area and pore size distribution	Templates are usually made from synthetic chemicals and can be costly for large‐scale production at the commercial level

**Figure 2:**
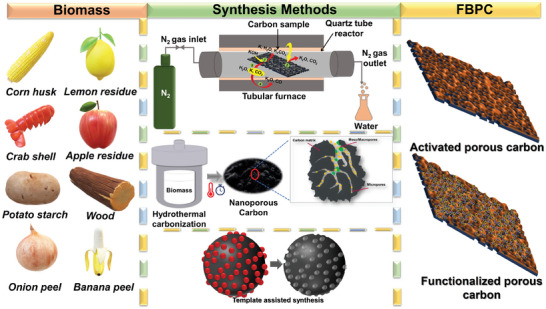
The schematic diagram shows various biomass precursors and synthesis methods for developing FBPCs.

A representation of the various synthesis methods including HTC, carbonization and activation, and template‐assisted is shown in **Figure** **2**.

### 2.1 Hydrothermal Carbonization (HTC)

HTC offers a quicker conversion of biomass into solid carbon at low temperatures as compared to conventional pyrolysis which requires high temperatures and a longer residence period. Moreover, HTC eliminates the need for pre‐drying the biomass which is a crucial step for the pyrolysis operation. The main controlling factors in HTC of biomass are the type of biomass feedstock and the reaction conditions such as temperature and residence time. The product of biomass HTC is often referred to as hydrochar and it contains an ample amount of surface functional groups including carboxylic acid and cyclic amines, and surface elements such as nitrogen and oxygen which can further be passed onto the porous carbons that can be prepared via chemical or physical activation of hydrochar. The FBPCs synthesized using a combination of hydrochar and carbonization/activation provide a high surface area in addition to the surface functional groups which manipulate the electrochemical properties and are crucial factors for electrochemical applications.^[^ ^29^ ^]^ Generally, HTC takes place in a closed reactor (autoclave) at elevated temperatures (usually between 180 °C and 250 °C) and pressures (typically 1–4 MPa)^[^ ^30^ ^]^ and involves a series of complex chemical reactions that can be summarized into three key stages:

#### 2.1.1 Hydrolysis and Depolymerization

At the start of the HTC process, biomass is introduced into the hydrothermal reactor along with water. In this high‐temperature and high‐pressure environment, water molecules act as both a solvent and a reactant. Under these conditions, the cellulose, hemicellulose, and lignin components of biomass undergo hydrolysis and depolymerization reactions. Water molecules attack the glycosidic bonds in cellulose and hemicellulose, breaking them down into smaller sugar molecules.^[^ ^31^ ^]^ Lignin, a complex phenolic polymer, undergoes cleavage reactions, leading to the release of phenolic compounds. These initial reactions result in the formation of soluble reaction intermediates, such as sugar‐derived compounds, organic acids, and phenolic compounds.^[^ ^32^ ^]^

#### 2.1.2 Carbonization and Condensation

As the temperature and pressure inside the autoclave continue to rise, the soluble reaction intermediates undergo further transformations. The dehydration and condensation reactions of these intermediates give rise to carbon‐rich species. These species gradually precipitate out of the aqueous phase and begin to form a solid carbonaceous material known as hydrochar. During this stage, carbonization reactions occur through the elimination of water and volatile organic compounds. This results in the formation of carbon‐carbon bonds and the development of an amorphous carbon structure within the hydrochar.^[^ ^33^ ^]^

#### 2.1.3 Functionalization and Porosity Development

One of the remarkable features of HTC is the concurrent functionalization of the hydrochar during its formation. This is primarily due to the presence of phenolic and carboxylic functional groups derived from the depolymerization of lignin and the subsequent reactions of phenolic compounds. Phenolic compounds in the hydrochar undergo oxidative reactions to form quinones and other oxygen‐containing functional groups. Carboxylic groups are also introduced through the oxidation of sugar‐derived compounds. These functional groups not only enhance the chemical reactivity of the carbon material but also contribute to its surface functionalities. Furthermore, HTC is also a facile ploy to incorporate metal species into the structure of hydrochar. For instance, HTC allows for the surface functionalization of hierarchical carbon derived from bamboo waste with cupric/cuprous oxide through the simple mixing of the Cu precursors with biomass.^[^ ^34^ ^]^ The HTC treatment also helped to preserve the heteroatoms created on the surface of carbon via a prior step of the calcination of bamboo waste. The surface functionalities facilitated rapid ion diffusion during electrochemical reactions and led to a reasonable specific capacitance.

**Figure 3:**
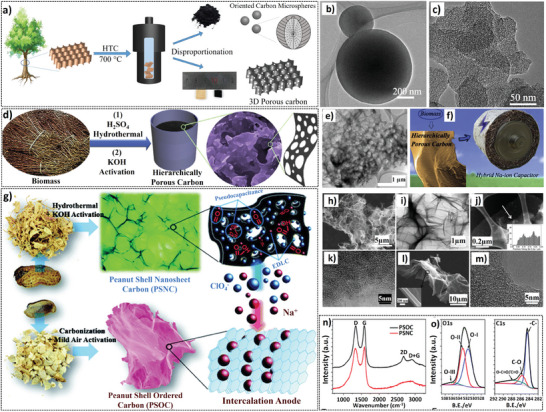
a–c) Schematic of the synthesis of OCMS and 3DPC from basswood via HTC treatment and their TEM images. Reproduced with permission.^[^ ^35^ ^]^ Copyright Wiley 2021. d–f) Synthesis of hierarchical porous carbon from biomass precursors via HTC treatment, its TEM and application as an electrode material for SIC. Reproduced with permission.^[^ ^36^ ^]^ Copyright Elsevier 2017. g–o) Synthesis schematic for PSNC and PSOC from peanut shell using hydrothermal treatment, and their structural evaluation using SEM, TEM, Raman and XPS data. Reproduced with permission.^[^ ^28b^ ^]^ Copyright Royal Society of Chemistry 2015.

Some of the recent highlights based on HTC of biomass have proven its benefits in improving the HIC performance. For instance, a unique high‐temperature hydrothermal disproportionation method (**Figure** **3**a) was employed to derive two different types of FBPCs from basswood including a 3D porous carbon (3DPC) (Figure 3b) and oriented carbon microspheres (OCMSs) (Figure 3c) which were then utilized as cathode and anode, respectively for a KIC device.^[^ ^35^ ^]^ The HTC process assisted in producing the two materials with desired features such as rich sp^3^ defects and micropores in 3DPC and specific orientation in OCMSs. The formation of the two different types of carbon materials was attributed to the successive decomposition of hemicellulose, cellulose, and lignin to form OCMSs followed by the dissolution of organic components to form 3DPC. This study proved that HTC treatment can effectively produce two different kinds of porous carbon materials from a single biomass source precursor. Moreover, the KIC fabricated with OCMS//3DPC exhibited high energy densities (140.7/65.2 Wh kg^−1^) at respective power densities of 643.8 and 6346.6 W kg^−1^. Another study reported the development of hierarchical porous carbons derived from the HTC treatment of various biomass precursors followed by chemical activation with KOH for their use as a cathode material for SIC (Figure 3d‐f).^[^ ^36^ ^]^ The HTC was performed using a given quantity of biomass and 1 M sulphuric acid (H_2_SO_4_) along with DI water, and it led to the hydrolysis of non‐crystalline hemicellulose and lignin components and reduced the crystallinity of cellulose in the biomass. The high temperature and pressure generated during HTC resulted in the formation of a carbon‐rich hydrochar which is a suitable starting precursor for KOH activation. The developed porous carbon after the activation was used as a cathode in SIC, which delivered energy density and power density of 169.4 Wh kg^−1^ at 120.5 W kg^−1^, respectively. Although the majority of research utilized H_2_SO_4_ for HTC treatment of biomass, nitric acid (HNO_3_) can also be a potential precursor for this procedure. For instance, low‐temperature HTC treatment with 0.5 M HNO_3_ followed by chemical activation with KOH was employed to prepare interwoven flake‐shaped porous carbon for utilization in SICs.^[^ ^28a^ ^]^ FBPCs containing oxygen surface functional groups were also prepared from peanut shells through HTC (2 M H_2_SO_4_) cum chemical activation (KOH) which were specifically denoted as interconnected nanosheet carbon (PSNC) (Figure 3g).^[^ ^28b^ ^]^ While PSNC was used as a cathode in SIC, the anode (peanut shell ordered carbon – PSOC) was also obtained from the same biomass through direct carbonization at 1200 °C. The microscopy results confirmed the 3D macroscopic amorphous morphology of PSNC and PSOC (Figure 3h–o). Raman spectroscopy detected the typical D and G bands of an amorphous carbon while XPS results confirmed that the materials are rich in carbon and oxygen elements.

### 2.2 Carbonization/Activation

Carbonization is the most sophisticated and convenient method of synthesizing carbons by using high temperature (400 to 600 °C) treatment of various carbon‐containing precursors under an inert environment.^[^ ^28c^ ^]^ Compared to HTC, wherein wet biomass can be utilized, carbonization generally requires biomass to be processed and dried as the first step. The carbons produced are termed biochar and depending upon the parent carbon source and the experimental conditions, it may or may not have significant porosity. Here again, activation, including physical and chemical is used to obtain porous carbons directly from biomass and/or biochar via high‐temperature (> 600 °C) treatment.^[^ ^22b^ ^]^ During the high‐temperature treatment, the presence of these activation agents results in carbon burn‐off and the evolved volatiles rupture the carbon walls to create porous structures with varying shapes and sizes.^[^ ^28d^ ^]^ Although porous carbons themselves are good candidates for several application fields, their efficiency can even further be enhanced through the introduction of various functional groups into their structure including heteroatoms and doping with metal atoms, etc.^[^ ^37^ ^]^ Carbonization cum activation offers the beauty of being utilized either in single or multi (mostly two‐step) step pathways, especially in the case of chemical activation. This primarily depends on the type of chemical activating agent and the nature of the biomass. For example, KOH and perhaps any potassium‐containing chemical activating agent offer more convenience of chemical activation with a high carbon‐containing biomass precursor such as biochar. This is due to a greater degree of redox reactions between KOH and biochar carbon which produces better results in terms of porosity and surface functionalization.^[^ ^22b^ ^]^ Hence such type of chemical activation is generally two steps; conversion of biomass into biochar followed by activation in the second step. On the other hand, chemicals such as zinc chloride (ZnCl_2_) mainly act as a dehydrating agent and are better used with original biomass that contains plenty of oxygen and hydrogen alongside carbon in biomass.^[^ ^38^ ^]^ Therefore, a one‐step carbonization cum activation is possible which reduced the time requirements for converting biomass into biochar in the first step and then proceeding with activation. This section particularly focusses on literature related to the synthesis of FBPCs using a combination of carbonization and activation and will cover some prominent examples from the recent literature to provide useful insights into this perspective.

**Figure 4:**
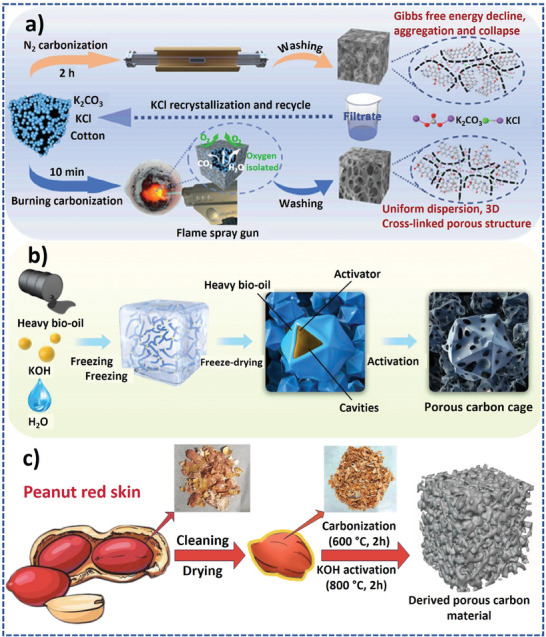
a,b) Variants of carbonization and activation using flame spray carbonization cum activation of biomass. Reproduced with permission.^[^ ^39^ ^]^ Copyright Elsevier 2023 and ice templating‐based carbonization and activation of heavy oil. Reproduced with permission.^[^ ^40^ ^]^ Copyright Elsevier 2023, and c) low‐cost biomass carbonization and activation of biomass for the synthesis of porous carbons. Reproduced with permission.^[^ ^41^ ^]^ Copyright Wiley 2023.

Although carbonization and activation are very well‐known procedures, there are recent developments that bring new insights into the synthesis of porous carbons. For instance, Jia et al. developed an idea to modify the carbonization procedure by shifting from the conventional slow pyrolysis approach to a much faster flame‐burning carbonization method (**Figure** **4**a).^[^ ^39^ ^]^

They took the biomass and activating agent (K_2_CO_3_ + KCl) in a crucible and heated it in a direct flame for a few minutes before washing the materials with HCl and water. This method produced porous carbons with a 3D interconnected porous hierarchical structure but with a lower surface area (1121 m^2^ g^−1^) which was in complete contrast to a broken porous structure and a higher surface area (1509 m^2^ g^−1^) obtained with the conventional carbonization procedure carried out in a tubular furnace at 900 °C. However, the hierarchical structure of the new type of carbon eventually led to a better performance of the material as a supercapacitor electrode (367 F g^−1^ at 0.5 A g^−1^ in a 6 M KOH as an electrolyte) as compared to the one synthesized using tubular furnace (248 F g^−1^ at 0.5 A g^−1^ using 6 M KOH as an electrolyte). In another example, Xiao et al. used an ice‐templating strategy to produce cage‐type porous carbon from the precursor of heavy oil through chemical activation with KOH (Figure 4b).^[^ ^40^ ^]^ The mixture of KOH solution and heavy oil was refrigerated and freeze‐dried, and the freeze‐dried material was then subjected to carbonization cum activation at 700 °C under argon flow. During refrigeration, the formed ice separates the heavy oil molecule leading to easy permeation of the KOH and the freeze‐drying removes the ice to create a templated structure. The hollow cavities created by ice were still preserved after the activation of the freeze‐dried material to produce porous carbons. Such a templated porous structure was conducive to achieving good performance in symmetric supercapacitors and zinc ion hybrid capacitors.

Biomass is a low‐cost raw precursor for use in carbonization and activation to produce porous carbons. Li et al. demonstrated that peanut red peels, which otherwise is a waste, as a low‐cost precursor can be carbonized and activated with KOH to form a high surface area porous carbon that is a suitable material as a cathode for a zinc ion hybrid supercapacitor (Figure 4c).^[^ ^41^ ^]^ Similarly, other low‐cost and waste biomass precursors such as peanut shell,^[^ ^42^ ^]^ hemp residue,^[^ ^43^ ^]^ coconut shell,^[^ ^44^ ^]^ and pistachio shell,^[^ ^45^ ^]^ among several others have also been reported for the synthesis of porous carbons via the process of carbonization and activation.

Biomass inherently contains heteroatoms such as oxygen and nitrogen which can be passed onto the synthesized porous carbon during carbonization and activation. Moreover, such heteroatoms can be easily doped externally into the porous carbon support as well to enhance their application performance. For example, N‐doped porous carbon can be obtained from the litchi shell by modification with urea and activation with KOH.^[^ ^46^ ^]^ Similarly, lignin can be modified with 3‐chloro‐2‐hydroxypropyl ammonium chloride and activated with potassium carbonate or zinc oxalate to produce N‐doped porous carbons.^[^ ^47^ ^]^ Surface oxygen groups can be introduced in lignite‐derived porous carbon by treating it with HCl washing and oxidation using H_2_O_2_.^[^ ^48^ ^]^ Moreover, porous carbons derived from biomass can easily be modified/accommodated with more than one heteroatom. For example, nitrogen and sulfur co‐doping can be achieved via modification of a porous carbon derived from *Amygdalus davidiana* shells‐based biomass with methylene blue at 200 °C.^[^ ^49^ ^]^ Nitrogen and oxygen self‐doped porous carbons can also be synthesized from *Cistanches herba* residues via KOH activation.^[^ ^50^ ^]^ The amount of data published in this field of FBPCs is enormous and a multitude of porous carbons synthesized using carbonization and activation to induce porosity and surface functionalization in porous carbons are reported frequently. Nevertheless, carbonization alone or in combination with activation is a well‐established technology to produce porous carbons from biomass with desired characteristics.

**Figure 5:**
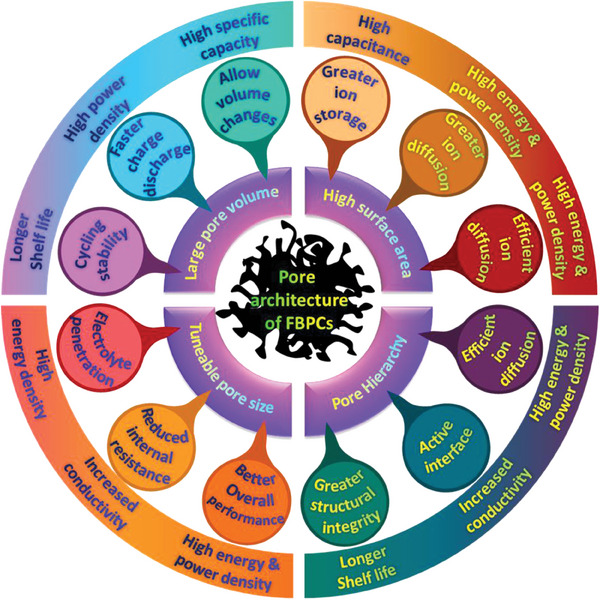
The effect of the pore architecture on various FBPCs material attributes and the performance factors of HIC.

As mentioned in this section, when designing FBPCs, it is crucial to maintain the porosity of the material as it is a dominant factor that influences the performance of the HICs.^[^ ^51^ ^]^ Higher porosity in FBPC stands for a higher surface area which is significant for the adsorption and desorption of the ions by providing an active interface area between the electrolyte and the electrode. A high surface area can originate from within the micropores and/or mesopores. The micropores provide an efficient pathway for ion diffusion, whereas mesopores contribute to effective and higher mass transport. A large pore volume is another important consideration and it stands for ample space for accommodating volume expansion during electrochemical reactions. A higher surface area would allow for the storage of a larger number of ions which leads to better HIC performance.^[^ ^52^ ^]^ Porosity also determines the diffusion of ions; higher porosity will induce a greater diffusion of ions in the supercapacitor component of HIC leading to a faster charge and discharge and hence higher power density, whereas it will also help in achieving a higher energy density in the battery component via efficient ion diffusion. Another critical factor is the penetration of the electrolyte into the electrode materials and the presence of micro and meso‐hierarchical porous structures can facilitate the process leading to better rate capability and power output.^[^ ^53^ ^]^ In the case of the supercapacitor component in HICs, it is worth noting that the internal resistance of the electrode material should be minimal which is highly likely to be achieved in materials with a great degree of interconnected porous structures. From a battery point of view in HICs, the porosity of FBPCs could help in facilitating the volumetric changes occurring during the charging and discharging cycles. This criterion ensures that the structural integrity of the electrode material is maintained for a longer period of cycling time. **Figure** **5** illustrates all the possible performance factors for HICs that are controlled by the porosity of the FBPCs. For example, the high surface area in porous carbons allows for greater storage, and higher and more efficient diffusion of ions whereas pore hierarchy dictates the structural integrity, active interface, and efficient ion diffusion. On the other hand, pore size controls electrolyte penetration, internal resistance while the specific pore volume accounts for cycling stability, accommodating volume changes and faster charge discharge.

Hard carbons like FBPCs are proven materials for the electrode of HICs and a good balance between the levels of functionalization and porosity can bring out the best possible results to achieve a high electrochemical performance. FBPCs, owing to their porous structure and other accompanying characteristics such as good thermal, chemical and mechanical stability, low‐cost manufacturing, and environmentally friendliness make them stand‐out materials for every category of monovalent and multivalent cations that are currently being explored for HICs.

Overall, porosity or the pore architecture of the FBPC‐based electrode material is a significant factor that influences the ion sorption, ion diffusion, power density of the supercapacitor component, electrolyte penetration, energy density of the battery component, cycling stability and the overall performance of the HICs. Therefore, FBPC with high porosity at the desired configurations can help achieve better performance in HICs and a good balance between the two components of the HICs.

Increased porosity in FBPCs can directly impact the energy density in HICs and is a result of several factors. Increased surface area leads to ample active sites for electrochemical reactions, which can lead to higher capacitance and hence higher energy density. Higher porosity in FBPCs improves ion diffusion which results in lesser resistance and improved electrochemical performance and energy density. The porous structure is also favorable for better penetration of electrolyte into the electrode for maximum utilization of the electrode to ensure obtaining high storage capacity and high energy density. Porosity also provides faster ion diffusion improving the rate capability, reducing energy loss, and hence delivering high energy density. The high surface area and excellent porosity in FBPCs allow for easy adsorption of metal ions onto the surface of electrode material.^[^ ^54^ ^]^ Perhaps the higher adsorption rate is made possible through efficient ion movement and easy access to the electroactive sites which collectively increases conductivity during the charge storage process.^[^ ^55^ ^]^ The high surface area in FBPCs is also an appealing factor in maximizing the electric double‐layer capacitance,^[^ ^56^ ^]^ ensuring remarkable charge storage and an increase in overall capacitance.^[^ ^57^ ^]^ Porosity is highly significant, in particular, for ZICs and SICs in improving their gravimetric and volumetric energy densities.^[^ ^58^ ^]^ For KICs, the use of FBPC results in enhanced capacity retention and cyclic stability. To illustrate the effect of porosity on energy density, KOH activation‐based *Areca Catechu* sheath‐derived porous carbon (ASICKOH) was employed for ZIC fabrication.^[^ ^59^ ^]^ ASICKOH showed a higher mesopore volume as compared to the non‐porous carbon, which contributed towards higher energy density in ASICKOH for ZIC. Hierarchical porosity can lead to enhanced K^+^ adsorption sites and consequently increased energy density in KICs.^[^ ^60^ ^]^ Graded porosity can cause easier penetration of electrolytes into the bulk of carbon even at high current density.^[^ ^61^ ^]^ The increase in energy density due to high surface area has also been recorded for LICs.^[^ ^62^ ^]^

### 2.3 Template‐Assisted Synthesis

The method of templated synthesis for FBPCs holds great promise in creating materials with precisely controlled pore structure and morphology. The controlled porosity and morphology of FBPCs are achievable by selecting an appropriate template and carefully manipulating the synthesis conditions. Various templating strategies, such as soft templating, self‐templating, hard templating, and salt templating, are employed in synthesizing FBPCs, providing versatility in tailoring their properties.^[^ ^9^, ^63^ ^]^ Zheng and co‐workers integrated soft templating and hydrothermal methods to develop mesoporous biochar using F127 (polyethylene polypropylene glycol) as the structure‐directing surfactant, and biomass batatas powder as a source of carbon.^[^ ^64^ ^]^ Implementing a soft templating strategy yielded a remarkable increase in specific surface area and pore volume due to the controlled self‐assembly of precursor molecules around the soft template, creating a network of interconnected pores within the carbon material. Likewise, self‐templated hierarchically porous carbon (HPC) through the activation of hard and soft carbon precursors containing rice husks and Pitch was developed by Xue et al.^[^ ^65^ ^]^ When subjected to gradual heating and activation, the soft Pitch is introduced into the gaps of the rice husk, resulting in the creation of a 3D interconnected porous structure. The inclusion of rice husks in this process plays a crucial role in generating internal pores within the carbon blocks due to the self‐templated effect and also increases the accommodation of oxygen content (9.07 at%). It was demonstrated that the soft‐hard templating method enhanced the electrical conductivity, increasing the amount of Pitch that lowered the defects while activation and have more ordered graphitic structure.

**Figure 6:**
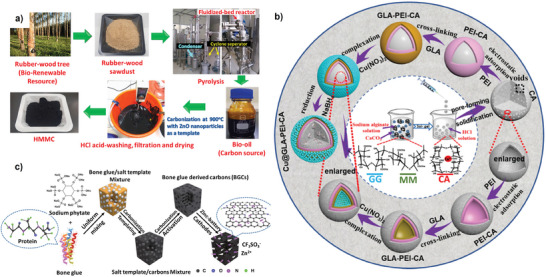
a) Schematic representation of the resource collection of rubberwood sawdust and its use in the synthesis of the HMMC. The rubberwood‐derived bio‐oil was further carbonized with ZnO NPs. The template was then removed by washing it with HCl and deionized water. Finally, the biomass‐derived HMMC was obtained after drying in an oven. Reproduced with permission.^[^ ^67^ ^]^ Copyright Elsevier 2021. b) Schematically representing the synthesis procedure of Cu@GLA‐PEI‐CA hydrogel beads. Reproduced with permission.^[^ ^70^ ^]^ Copyright Elsevier 2018. c) Schematic illustrating BGCs preparation procedure. Reproduced with permission.^[^ ^74^ ^]^ Copyright Springer 2021.

Other than soft and self‐templating methods, the hard templating method is also a versatile approach for synthesizing FBPCs with well‐defined pore structures and tunable textural properties employing ordered inorganic porous solids (zeolites, mesoporous silica, clays, etc.) as templates. The use of hard templates helps in achieving an ordered mesoporous structure, where the pores are organized in a regular pattern. This ordered arrangement can benefit various applications, including catalysis, gas storage, and energy storage.^[^ ^28d^ ^]^ Although hard templating is potentially viable and efficient, developing porous carbon with templates like silica, clay, and zeolites is considered expensive and difficult to use on a large scale as these templates need to be removed using corrosive chemicals such as HF or NaOH.^[^ ^66^ ^]^ Alternatively, biomass resources are cheap, abundant, and renewable, and their utilization as carbon sources and constructing porous carbon with new emerging hard templates (ZnO, CaCO_3_, NaCl, MgO) could produce controllable structures displaying higher performances in energy storage and adsorption^[^ ^63^ ^]^ applications. Herein, we discuss some selected works on emerging hard templates and utilizing FBPCs for energy storage applications. Benefiting from the advantages such as low cost, easy removal of templates, and imperative physical and chemical properties, Lin et al. developed hierarchical micro/mesoporous carbons (HMMCs) using rubberwood oil as a sustainable biomass carbon source and zinc oxide nanoparticles (ZnO NPs) as a template.^[^ ^67^ ^]^ In the process illustrated in **Figure** **6**a, rubberwood underwent treatment in a fluidized‐bed reactor at a high temperature of 500 °C to produce bio‐oil. The extracted bio‐oil was mixed with ZnO NPs and subjected to carbonization at 900 °C to fabricate HMMC. The resulting material exhibited spherical nanopores with an impressive specific surface area of 1365 m^2^ g^−1^, including a micropore area of 530 m^2^ g^−1^, which was further hybridized with Li_4_Ti_5_O_12_ (LTO) to serve as an electrode material for LICs. ZnO NPs serve a dual purpose in synthesizing BPCs, functioning both as a template and an activator.

Likewise, Cho and co‐workers developed hierarchical porous carbon (HPC) using CaCO_3_ as a template and bio‐oil resin produced using rubber wood sawdust, formaldehyde, and 50% KOH solution.^[^ ^68^ ^]^ The 3D honeycomb morphology with a mesoporous structure offered biomass‐derived HPC which exhibited a high specific surface area of 1604.9 m^2^ g^−1^ with a pore size of < 0.9 nm. The transformation of bio‐oil to resol resins favored the formation of HPC with higher product yield and improved surface area compared to HMMC. Also, they demonstrated that manipulating the content of the CaCO_3_ template influences the development of porous structures in resulting carbon materials, leading to hierarchical pores with a broadened range of size. Likewise, Qin and co‐workers developed a method to create defect‐rich nitrogen‐doped porous carbon by employing CaCO_3_ as a hard template and sodium alginate (SA) as the carbon source.^[^ ^69^ ^]^ They initiated the process by introducing a mixture of urea, CaCO_3_, and SA into a 0.05 M HCl solution, resulting in the formation of urea‐modified calcium alginate beads. Through subsequent pyrolysis and etching, a 3D architectured porous structure with macropores displayed a specific surface area of 1117 m^2^ g^−1^ and pore size of ≈4.4‐6.5 nm. In this process, the macropores created by SA during the solidification into the hard template accommodated more urea and contributed to developing an N‐doped porous structure.

In another report, Wang et al. employed a modified method to synthesize copper nanoparticles supported glutaraldehyde‐crosslinked polyethyleneimine‐modified calcium alginate beads (Cu@GLA‐PEI‐CA) utilizing CaCO_3_ as the hard template.^[^ ^70^ ^]^ A schematic representation showing the synthesis procedure is depicted in Figure 6b. The dual function of CaCO_3_ NPs to act as both a hard template and a cross‐linking agent suggests a synergistic effect. The combination of these roles led to the formation of 3D interconnected honeycomb‐like porous structures with a surface area of 139 m^2^ g^−1^ and pore sizes of ≈19 to 23 nm. Importantly, the diameter and even distribution of Cu NPs within the structure can be finely tuned by adjusting the concentrations of the CaCO_3_ in the NP solution. Xi et al. developed a new in situ templating method to produce FBPCs using alginate and FeCl_3_.^[^ ^71^ ^]^ The process involves creating ferric alginate hydrogel beads, followed by freeze‐drying and carbonization at 700 °C making Fe‐SA‐C. During carbonization, Fe(OH)_3_ nanoparticles embedded within the hydrogel act as a hard template, facilitating the formation of 3D interconnected porous carbon structures. Fe‐SA‐C materials displayed surface areas in the range of 645.6 m^2^ g^−1^ to a high of 902.8 m^2^ g^−1^ while altering the mass of FeCl_3_ in the synthesis process (0.5 to 2 g). Moreover, the conversion of Fe_2_O_3_ nanoparticles during heat treatment facilitates the formation of rich pores (2–10 nm) with high specific surface area and the formation of highly graphitized carbon with improved electrical conductivity.

The salt template method represents an alternative approach for synthesizing FBPCs, where biomass can be a sustainable and renewable carbon source, making this approach attractive from an environmental perspective. In this method, the molten salt (ZnCl_2_, KCl, NaCl, LiCl, sodium phytate) acts as a template, reaction medium, and flux for creating nanopores, typically converting biomass‐derived precursors into functional materials. Molten salts have relatively low eutectic points compared to other activation methods, enabling the activation process to occur at lower temperatures, saving energy, and reducing the potential for degradation of the carbon precursor. After activation, it can be easily removed from the product using water or diluted acid, isolating the final product much easier and more efficiently than other methods requiring harsh chemicals or high‐temperature treatments. The as‐prepared materials display a porous structure offering several benefits, including a high surface area, good electrical conductivity, and efficient mass transport. These properties make the resulting hierarchically porous carbons ideal for various applications, such as energy storage, catalysis, and environmental remediation.^[^ ^72^ ^]^

For instance, Zou and co‐workers illustrated the fabrication of N‐rich porous carbon (NDPC) from sugar cane bagasse serving as a biomass carbon precursor and a combination of ZnCl_2_, KCl, and urea as the molten‐salt template.^[^ ^73^ ^]^ The amalgamation of these salts functions as a low‐temperature solvent for blending the biomass, creating a template that subsequently serves as an activator to induce the development of a porous structure. NDPC exhibited a structure characterized by ribbon‐like particles intricately interwoven, resulting in an interpenetrated porous configuration. This arrangement conferred a notably high specific surface area of 1505.9 m^2^ g^−1^, accompanied by pores measuring 2.43 nm in size. The introduction of KCl positively influenced the overall pore volume, demonstrating a twofold enhancement compared to materials with lower amounts of KCl. In another report, Hu et al. introduced a modified method to produce porous carbon from sweet potatoes, involving the utilization of ZnCl_2_ and NaCl as salt templates during the carbonization process, followed by additional activation steps.^[^ ^72a^ ^]^ Although the derived activated carbon (AC) displayed irregular morphology, the template‐directing role featured a significantly high surface area of 3424 m^2^ g^−1^ with abundant micropores and nitrogen‐functional groups. Similarly, Zhang et al. successfully employed a molten metal chloride salt template method to create N/S‐doped porous carbon nanosheets (PCNS) with advantageous 3D structures and functionalities, using ginkgo leaves as a biomass source.^[^ ^72b^ ^]^ Compared to the impressive 3424 m^2^ g^−1^ surface area achieved using ZnCl_2_/KCl templates in AC, the 0.75PCNS_800_ sample prepared with CaCl_2_/KCl displayed a significantly reduced surface area of only 395.1 m^2^ g^−1^. The observed features highlight the necessity of exploring alternative templates/activating agents instead of CaCl_2_‐KCl to achieve optimal performance. On the other hand, Fan et al. introduced a co‐assisted carbonization technique involving the templating and activation of protein‐rich bone glue biomass (Figure 6c). This process utilizes sodium phytate as the salt template and NaOH as an activating agent to produce carbons derived from bone glue (BGCs).^[^ ^74^ ^]^ The template sodium phytate facilitates the creation of macro/meso‐porosity and promotes maximum utilization of NaOH in the activation process. In addition, the carbon source bone glue is a protein, comprising of polypeptide chains that incorporate nitrogen and oxygen. The activation process, assisted by sodium phytate template, securely retains the heteroatoms within the carbon structure, showcasing them as inherent FBPCs. **Table** **2** depicts various FBPCs, their synthesis method and the corresponding properties such as morphology, surface analysis, modification strategies, and related application.

**Table 2:** Comparing FBPCs based on their synthesis method and the corresponding properties.

Material	Parent biomass	Synthesis method/ conditions	Morphology	Textural features		Heteroatom/ Metal dopant	Type of application	Reference
				Surface area [m^2^ g^−1^]	Pore size [nm]			
HMC‐800	Batatas	Soft templated/ hydrothermal	Spheres	286.3	4.3	–	Adsorption	[64]
RH_1_SP_1_‐HPC	Rice husk and Pitch	Self‐templated	3D	2996	0.5–4	O	Supercapacitor	[65]
DNPC	Sodium Alginate	Hard templated (CaCO_3_)	3D	1117	4.4–6.5	N	Supercapacitor	[69]
Cu@GLA‐PEI‐CA	Sodium Alginate	Hard templated (CaCO_3_)	3D‐honeycomb structure	139	15.8–23.04	Cu	Catalysis	[70]
Fe‐SA‐C	Alginate	Hard templated (Fe(OH)_3_)	3D	902.8	2.0–10	Fe	Supercapacitor	[71]
NDPC	Sugar cane bagasse	Molten‐salt templated/ ZnCl_2_‐KCl	Ribbon‐like intertwined	1505.9	2.43	N	LICs	[73]
AC	Sweet potato	ZnCl_2_‐NaCl templated/KOH activation	Irregular	3434	1.0–3	N	ZICs	[72a]
PCNS	Ginkgo leaf	CaCl_2_‐KCl templated	3D sheets	395.1	0.5–2	N, S, O	Supercapacitor	[72b]
BGCs	Bone glue	Sodium phytate templated/ NaOH activation	Macro porous honeycomb	3657.5	2.5–5.7	Zn	ZICs	[74]
HPC‐MnO	Lichee shells	Chemical activation	Flake like structures	1486	2	MnO	Supercapacitor	[75]
PCMs	camellia oleifera branches	Carbonization and activation	Coiled sheets	938.5	2.0–3	NH_4_Cl	Supercapacitor	[76]
P‐AC/Co_3_O_4_	peanut shells	Carbonization and activation	Channels	1535.9	<50	Co_3_O_4_	Supercapacitor	[77]
CuOx@C	bamboo leaves	Carbonization and hybridization	Lamellar	325.25	2.39	CuO	Hybrid like capacitor	[34]
HSAC	peanut shells	Hydrothermal method	Bulk	1279	2.2	–	Supercapacitor	[78]
NAC	Rice straw	Carbonization and activation	Porous	2537	1.2	Melamine	Supercapacitor	[79]
PBC	pine petals	Hydrothermal carbonization	Honeycomb	1726.3	5	NiCl_2_, H_2_C_2_O_4_	Supercapacitor	[80]
WSC0	Sawdust	Hydrothermal carbonization	Stacked	1185	3.27	KOH	Supercapacitor	[81]
PC	Pinewood, candlenut, cedarwood	Carbonization and activation	Bulk	1688	3.98	H_3_PO_4_, KOH	Supercapacitor	[82]
CPC	Chitosan	Chemical activation	Porous/ spongy	2278	0.56,0.73	Potassium citrate	CO_2_ adsorption	[37]
PSR‐4	Peanut red peels	Carbonization & activation	Porous carbon blocks	2072	3.16	C, O (KOH)	ZIC	[41]
PAC‐1.5K_2_CO_3_‐750‐0.5	Peanut shells	Alkali activation	Porous carbon	669	2.57	K_2_CO_3_	CO_2_ capture	[42]
AC	Hemp residue	H3PO4‐assisted hydrothermal carbonization	Porous carbon	1630	–	N doping (NH_4_NO_3_, pyridine)	Supercapacitor	[43]
AC‐13	Coconut shells	Carbonization & activation	Porous carbon	2410	3.35	–	Supercapacitor	[44]
GPB4‐1000	Pistachio shell	Activation and graphitization	Porous/ spongy	1673	0.52, 4	–	CO2 capture and LIBs	[45]
N‐LPC	Litchi shell	Activated calcination route	Porous carbon	211.1	–	N	Zn‐ Iodine batteries	[46]
PLC‐Si	Lignin	Quaternization and carbonization	Honeycomb porous structure	1139	–	N	Supercapacitor	[47]
ZCAC	Amygdalus davidiana shells	Activation	Porous carbon	1734.58	4.35	O, N, S	Supercapacitor	[49]
CHC	Cistanches herba	Pre carbonization and activation	Porous inner surface	2767.2	–	N, O	Supercapacitor	[50]

**Note**: Hydrothermal mesoporous biochar (HMC); Rice husk soft pitch hierarchical porous carbon (RH SP‐HPC); Defect rich N doped porous carbon (DNPC); Glutaraldehyde solution (GLA); calcium alginate (CA); Sodium alginate (SA); Nitrogen doping porous carbon (NDPC); Activated carbon (AC); Porous carbon nanosheet (PCNS); Bone glue derived carbon (BGC); Hierarchically porous carbon/manganese monoxide nanosheet (HPC‐MnO); Nitrogen‐doped porous carbon materials (PCMs); porous activated carbon/Co_3_O_4_ (P‐AC/Co_3_O_4_); Hydrothermally synthesized activated carbon (HSAC); nitrogen doped Acs (NAC); porous biomass derived carbon (PBC)Sawdust derived porous (WSC0); Porous carbon (PC); Chitosan derived porous carbon (CPC); Peanut red skin waste derived carbon (PSR); Activated carbon (AC); Graphitized pistachio shell biochar (GPB); Nitrogen doped litchi shell derived porous carbon (N‐LPC); Nitrogen doped lignin derived porous carbon (PLC); *Amygdalus davidiana* shells derived carbon (ZCAC); *Cistanche Herba* derived carbon (CHC).

## 3 Types of HICs and the Usage of FBPCs as Electrode Materials

FBPCs exhibit improved electrochemical performance in HICs because of their predominantly EDLC storage mechanism. The properties of the FBPC‐based electrode will vary with respect to the biomass precursor, its concentrations and synthesis method and parameters. Thus, developing electrode material with high charge storage capacity is of prime importance in HICs.^[^ ^83^ ^]^ Based on the valence of the metal cations, HICs are classified into two categories: (i) Monovalent HICs which involve LICs, SICs and KICs, and (ii) Multivalent HICs including ZICs, Mg‐based HICs (MICs), Al‐based HICs (AICs) and Ca‐based HICs (CICs).^[^ ^13^ ^]^ According to the electrode configuration, HICs are categorized based on whether FBPC is employed as a cathode, anode, or dual electrode. Recent updates on FBPC‐based HIC device applications are discussed below.

### 3.1 Monovalent HICs

#### 3.1.1 Lithium Ion Capacitors (LICs)

LICs are the most researched form of HICs and were first developed by Amatucci who introduced LTO as an anode and activated carbon as a cathode material for their LIC.^[^ ^84^ ^]^ The inherent disparity in kinetics and capacity between the cathode and anode electrodes of the LICs is often an issue which could be addressed by creating an anode material that facilitates rapid diffusion of lithium ions and efficient charge transfer, paired with a higher capacity cathode.^[^ ^85^ ^]^ On the other hand, the electrochemical properties of capacitor‐type cathode material in LICs are primarily influenced by the accessible area of ions.^[^ ^86^ ^]^ Most importantly, the ionic radii corresponding to the monovalent charge carrier are of great importance for detailed information on the ion intercalation/deintercalation mechanism.^[^ ^87^ ^]^ The cathode materials associated with LICs using FBPCs have witnessed thorough exploration due to their porous structure which enables rapid adsorption/desorption as well as exceptional surface area for Li^+^ ion storage.^[^ ^88^ ^]^ FBPCs are reported to function both as anode and cathode and also as dual electrode material for LICs. Some of the prominent instances in this regard will be discussed in the following subsections.

##### FBPC Based Dual Carbon Electrodes for LICs

**Figure 7:**
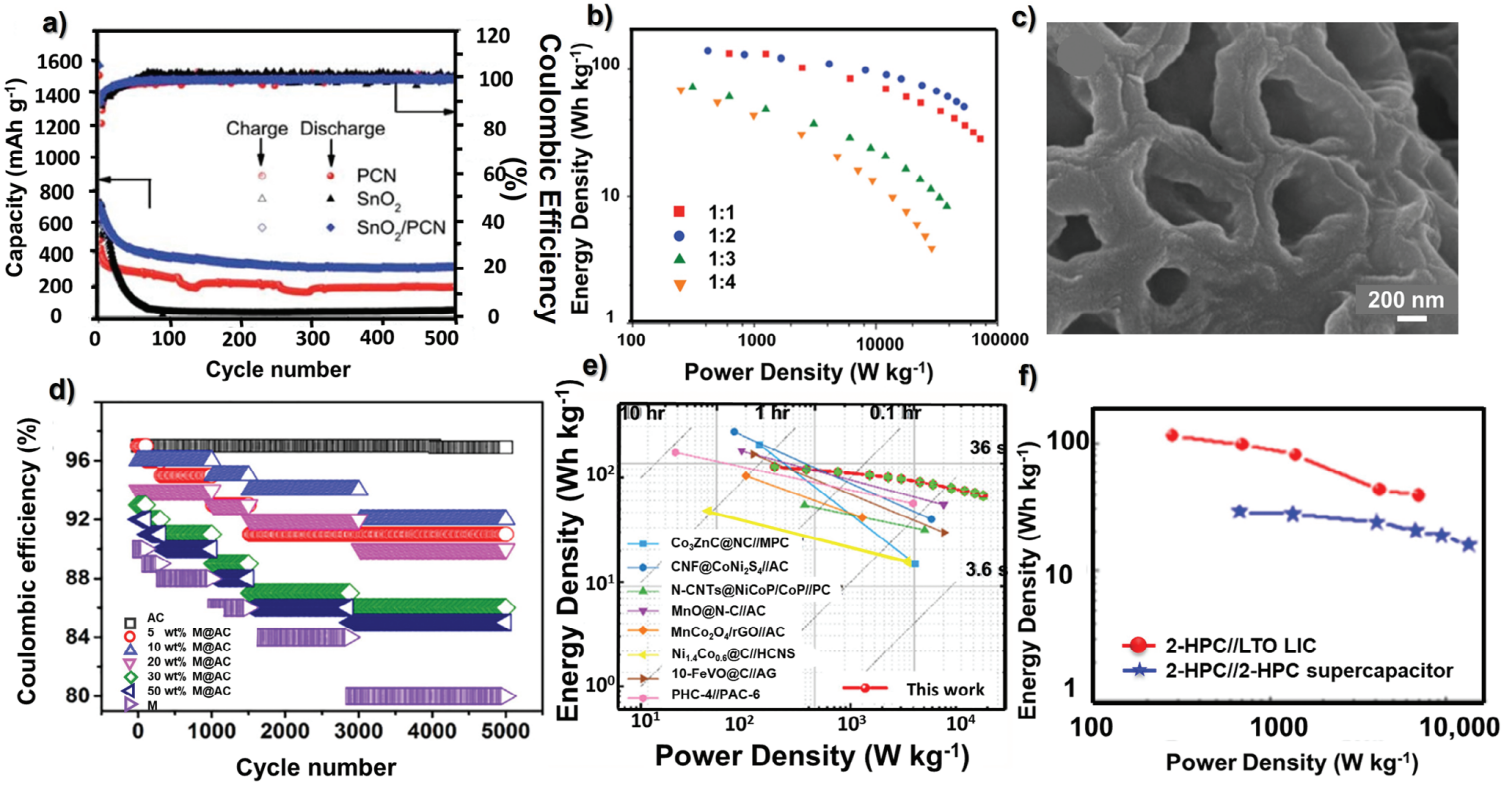
a) Cyclic stability test and corresponding coulombic efficiency for PCN, SnO_2_ and SnO_2_/PCN at 1 A g^−1^ (500 cycles), (=b) Ragone plot showing the electrochemical performance of LIC with various anode to cathode mass ratios. Reproduced with permission.^[^ ^92^ ^]^ Copyright Royal Society of Chemistry 2021. c) SEM images of functional group‐rich FBPC. Reproduced with permission.^[^ ^93^ ^]^ Copyright MDPI 2021. d) Coulombic efficiency of LIC based on AC@Mn_2_O_3_ for 5000 cycles at 5 A g^−1^. Reproduced with permission.^[^ ^97^ ^]^ Copyright Elsevier 2020. e) Ragone plot of NiCoP//WPBC‐6. Reproduced with permission.^[^ ^98^ ^]^ Copyright Elsevier 2024. (f) 2‐HPC//2‐HPC and 2‐HPC//LTO LICs. Reproduced with permission.^[^ ^68^ ^]^ Copyright MDPI 2022.

FBPCs are exceptional materials as these can be utilized as the electrode materials for either of the electrodes in LICs. For instance, graphene carbon composite developed from treating coffee waste and graphene oxide for LIC device application delivered around 100 Wh kg^−1^ energy density with a corresponding power density of 9000 W kg^−1^.^[^ ^89^ ^]^ The graphene addition results in enhancing the mechanical stability as well as the connection between the current collector and carbon matrix. FBPCs provide ample space for depositing functional transition metal oxides/hydroxides etc. thereby reducing agglomeration. The pseudocapacitive materials, such as MnO_2_, Fe_2_O_3_, NiO, Co_3_O_4_, and Ni(OH)_2_, exhibit superior capacity and redox activity compared to carbon materials, owing to the multivalent nature of transition metals.^[^ ^90^ ^]^ A dual carbon FBPC, derived from kapok fibre along with 2D MnO nanocomposite was employed for LIC device application.^[^ ^91^ ^]^ The MnO‐based carbon nanocomposite exhibited high surface area and thereby facilitated the Li‐ion insertion/extraction. The device delivered an energy density of 100 Wh kg^−1^ and a power density of 83 W kg^−1^. In another instance, an anode comprising pre‐lithiated SnO_2_ anchored on porous carbon nanosheets (SnO_2_/PCN) and a cathode made up of bare porous carbon (PCN) were fabricated and tested for their electrochemical behaviour in LICs.^[^ ^92^ ^]^ Anode exhibited excellent cyclic performance of 313 mAh g^−1^ after 500 cycles due to the synergetic effect of SnO_2_ and a conductive carbon substrate (**Figure** **7**a). The electrochemical performance of the LIC (SnO_2_/PCN//PCN) fabricated with various anode‐to‐cathode mass ratios is depicted in the Ragone plot (Figure 7b). The optimized hybrid system delivered energy densities of 138 and 51 Wh kg^−1^ at power densities of 416 W kg^−1^ and 53k W kg^−1^, respectively.

Another study reported pre‐lithiated Fe_3_O_4_@ carbon‐based anode and activated carbon‐based cathode LIC with energy densities of 140.6/ 52.8 Wh kg^−1^ at power densities of 200 W kg^−1^/10k W kg^−1^.^[^ ^93^ ^]^ The functional groups were rich in carboxyl and hydroxyl groups over the FBPC making its surface rough and allowing FeOOH growth over the carbon surface easily. SEM image of the corresponding FBPC is shown (Figure 7c). The values of energy density and power density obtained in these FBPCs are exceptional, however, a relative comparison between the two studies cannot be easily established due to variations in several parameters including the nature of biomass used, structural and physico‐chemical properties of the porous carbon and the type of chemical species used for the modification. The energy density values are not far from commercial Li‐ion batteries (>200 Wh kg^−1^) and the power densities are either comparable to or almost five times higher than commercial capacitors (10k W kg^−1^).^[^ ^7a^ ^]^ Beyond the metal oxide and carbon composites, the incorporation of metal sulfides into FBPCs has garnered attention, leveraging their enlarged layered structure, redox variabilities, and high structural stabilities to provide additional storage sites. For instance, Wang et al. developed FBPC derived from bamboo powder and combined it with nano‐ ZnS (CZS) for fabricating LIC device.^[^ ^94^ ^]^ The device with FBPC‐based composite and N, S doped porous carbon as anode and cathode delivered an energy density of 158 Wh kg^−1^ at a power density of 10k W kg^−1^. The tight integration of FBPC and ZnS balances the capacity and improves the electrochemical performance. The elemental mapping showed the presence of ZnS nanoparticles along with nitrogen.

Heteroatom‐doped FBPCs have frequently been explored for LICs. For example, Zeng et al. developed N‐doped high surface area FBPC from a different precursor of cloth fiber and reported an energy density of 186.31 Wh kg^−1^ at a power density of 225 W kg^−1^.^[^ ^95^ ^]^ An effective method of dual system activation was employed to create nitrogen‐doped biomass‐derived carbon with hierarchically porous architecture (HNBC), where a significant quantity of nitrogen doping can increase conductivity and make it easier for charge transfer during charging and discharging. The constructed asymmetric LIC provided a high‐power density of 225 W kg^−1^ and an energy density of 186.31 Wh kg^−1^, along with excellent cycling stability.

Apart from single‐atom doping, co‐doping with two heteroatoms is also an approach widely utilized for devising electrode materials for LICs. A dual carbon LIC device developed by constructing a cathode and anode made of unique N, S‐co‐doped porous carbon (NS‐ACSC) and N, S‐co‐doped fractal‐like carbon nanoparticles exhibited excellent reversible ion storage capacity. The mass ratio‐optimized dual carbon NS‐CSC//NS‐ACSC (1:2) LIC device, which uses doped carbon electrodes, produced a high specific energy of 101.7 Wh kg^−1^ at 113 W kg^−1^, and offered 81% of its capacity after 7000 cycles at 2 A g^−1^. The impact on rate capability and good cyclic stability of the carbon‐based anode is explained due to the following factors: i) co‐doping of N and S heteroatoms in the carbon results in mitigating the volume changes during cycling of anodes and allowing faster Li^+^ ions insertion, ii) presence of heteroatoms that provides additional active sites and intrinsic defects on the edges and surfaces for reversible Li^+^ adsorption, enabling improved storage capacity.^[^ ^96^ ^]^ For instance, Sharma et al. used candle soot to develop high surface area N and S co‐doped FBPC which delivered an energy density of 101.7 Wh kg^−1^ at a power density of 113 W kg^−1^. These examples illustrate that either energy density or the power density in LICs can be heightened at the expense of each other, however, there is a rarity in the reported literature where both attributes are reported high simultaneously.

##### FBPC Based Cathode Materials for LICs

FBPCs can also be employed as a cathode material for LICs,^[^ ^99^ ^]^ which is largely possible due to their exceptional properties such as high surface area, abundant micropores, high electronic conductivity, high specific capacity, and layered porous structure. Jack fruit‐based activated carbon (JFAC) with a high surface area when utilized in conjunction with Co_3_O_4_ nanosheets as an anode, helped to deliver an energy density of 118 Wh kg^−1^ at 50 °C^[^ ^100^ ^]^ for LIC. The amorphous JFAC accounted for excellent EDLC and exhibited excellent capacity while managing the volume changes. The CV curves showed an abrupt surge in current at a higher potential due to the surface redox reaction of Co_3_O_4_. The study suggested that the combined effect of porous structure, 2D morphology of Co_3_O_4_ nanosheets and effective mass loading between the anode and cathode is crucial in improving the electrochemical performance of the LIC. In another instance, a palm kernel shell based on activated carbon (AC) was coated with a thin metal oxide film of Mn_2_O_3_ (AC@Mn_2_O_3_) and tested for LIC in a half‐cell configuration by using 1 M LiPF_6_ as an electrolyte.^[^ ^97^ ^]^ Besides the excellent EDLC behavior and electrochemical stability, the cycling stability was also good which revealed 98% and 97% capacity retention for AC and Ac@Mn_2_O_3_ respectively. Pure AC demonstrated exceptional coulombic efficiency of 97% after 5000 cycles (Figure 7d).

Another study highlights that the inherent disparity in kinetics and capacity between the LIC electrodes can be resolved by using in situ carbon‐doped surface unsaturated sulfur enriched cobalt disulfide at reduced graphene oxide (CoS_2_@rGO) aerogel as anode and crabapple leaves derived porous activated carbon as cathode materials.^[^ ^85^ ^]^ It was found that a potential range of 1.5–4.3 V is ideal for obtaining maximum specific capacity, reducing the capacity and kinetic mismatch between two electrodes. A LIC device based on a porous carbon cathode made up of coconut shell and CoF_2_ as an anode delivered good energy densities of 82/50 Wh kg^−1^ at corresponding power densities of 190 and 3800 W kg^−1^.^[^ ^101^ ^]^ Another instance reports a good performance in energy (127/67 Wh kg^−1^) and power densities (190/18 240 W kg^−1^) by using a bimetallic phosphide (NiCoP) and watermelon peel‐based porous carbon (WPBC‐6) as anode and cathode, respectively (Figure 7e).^[^ ^98^ ^]^ These two studies highlight that the variation in the performance of LICs can depend upon the type of FBPCs used as a cathode and further on the chemistry of the anode material. There are several other instances which highlight that FBPCs can be an excellent cathode material for LICs. Some of these include the use of resin (113.3 Wh kg^−1^ and 7031 W kg^−1^),^[^ ^68^ ^]^ and ginkgo leaves (118 Wh kg^−1^, 31.6k W kg^−1^)^[^ ^102^ ^]^ based porous carbon as cathode materials for LICs. Here, the resin‐based FBPC tested for LIC and supercapacitor (2‐HPC//LTO and 2‐ HPC//2‐HPC) and the overall energy density is higher for LIC than that of the corresponding supercapacitor device (Figure 7f). FBPCs have rarely been directly employed as anode for LICs, but as a support to hold onto other materials of interest and therefore it is not covered in this review.

#### 3.1.2 Sodium Ion Capacitors (SICs)

The SICs normally consist of Na ion‐based battery anode owing to a faradaic reaction and a supercapacitor cathode based on a non‐faradaic reaction.^[^ ^103^ ^]^ Just like LICs, addressing the kinetic imbalance between the positive and negative electrodes is crucial in SICs as well. One of the disadvantages of SICs when compared with LICs is the volume expansion due to the large ionic radii of Na^+^. To tackle the challenges associated with SIC, researchers are contemplating two primary strategies: shortening diffusion pathways through structural and morphological adjustments and incorporating a pseudocapacitive reaction mechanism for sodium storage via heteroatom doping. FBPCs are one of the prime candidates and have widely been reported as SIC electrodes in the existing literature.

##### FBPC Based Dual Carbon Electrodes for SICs

Cork‐derived carbon nanosheets proved to be good candidates as SICs dual electrode.^[^ ^104^ ^]^ The SIC device with cathode and anode fabricated with S‐doped carbon nanosheets and porous carbon sheets respectively from the same biomass used as dual carbon electrode delivered a high energy density of 141 Wh kg^−1^ and power density of 29k W kg^−1^. Likewise, utilizing sulfur‐doped carbon sponges as the anode and 3D porous carbon derived from gluten as the cathode led to an energy density of 72 Wh kg^−1^ and a power density of 24.2k W kg^−1^.^[^ ^105^ ^]^ The anode component demonstrates an impressive sodium ion storage capacity of around 524 mAh g^−1^ at 0.1 A g^−1^. The presence of sulfur in the anodic material proved crucial to achieving a high rate of faradaic redox reactions between sulfur and sodium which led to an increased capacity of 40–70% and 100–120% under lower and higher sodiation rates, respectively. The sustainable synthesis of biomass‐derived carbon electrodes with hybrid energy‐storage behaviors for use in high‐performance Na‐ion‐based batteries and hybrid capacitors was reported by Jin Niu et al.^[^ ^106^ ^]^ Gelatin, which contains a lot of C and N atoms, and phytic acid, which is rich in P and C atoms, were used as biomass precursors for heteroatom doping of two carbon compounds by separate customized procedures. The porous nanosheet morphology and heteroatom doping give the carbon electrodes battery‐capacitive storage properties, which results in their high electrochemical performance in half cells. The developed SIC exhibited high energy density (135.3 Wh kg^−1^), power density (16.1k W kg^−1^), and ultralong lifetime (88.6% of the initial capacity after 8000 cycles) due to the compatible kinetics of the cathode and anode.

A potato starch‐derived porous carbon (APSRC) was allowed to perform as both cathode and anode in SIC with promising energy density (76.4 Wh kg^−1^) and power density (150 W kg^−1^).^[^ ^90a^ ^]^ As compared to the previously discussed SICs fabricated with sulfur‐doped carbon as anode and porous carbon as the cathode, the power density, as well as the energy density of APSRC, is low which may be due to a lack of heteroatoms such as sulfur (14–27wt%,^[^ ^104^ ^]^ 13wt%^[^ ^105^ ^]^) and the low surface area. Jute‐based porous carbon proved to be an excellent precursor for an aqueous symmetric SIC delivering energy densities of 37.7 and 9.75 W h kg^−1^ at corresponding power densities of 785 and 7895 W kg^−1^, respectively.^[^ ^90b^ ^]^ In comparison, the same system in a non‐aqueous environment delivered an improved energy density of 60 Wh kg^−1^ but a lower maximum power density of 3570 W kg^−1^. The energy density can be further improved to 86 Wh kg^−1^ using asymmetric SIC.

**Figure 8:**
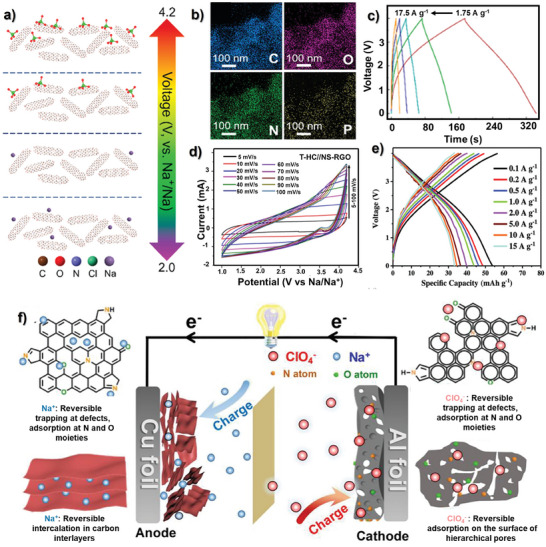
a) Schematic diagram showing energy storage mechanism, b) EDX elemental mapping of N‐PCNS‐KOH cathode and c) GCD curves of P, N‐HPCNS‐KCl/Ice||N‐PCNS‐KOH based SIC. Reproduced with permission.^[^ ^106^ ^]^ Copyright American Chemical Society 2020. d) Cyclic Voltammetry (CV) curves of T‐HC//NSRGO symmetric SIC device. Reproduced with permission.^[^ ^107^ ^]^ Copyright Energies 2023. e) Charge discharge curves of SIC using Na_2_Ti_3_O_7_ nanobelt/CNF anode and BPC/CNF cathode. Reproduced with permission.^[^ ^108^ ^]^ Copyright Wiley 2021. f) Charge storage mechanism of PSCS‐600//OPDN‐CTF‐A SIC. Reproduced with permission.^[^ ^109^ ^]^ Copyright Elsevier 2021.

A careful evaluation of the amount of the surface area and heteroatom doping affecting the performance in SICs reported by Wang et al. in their studies on carbon materials derived from gelatin and phytic acid.^[^ ^106^ ^]^ Generally, carbon material with a high surface area is employed as a cathode for getting high coulombic efficiency along with a low surface area anode. For instance, phosphorous and nitrogen‐doped materials developed using KCl and ice templating strategy (P, N‐HPCNS‐KCl/Ice) were used as anode and nitrogen‐doped materials activated with KOH (N‐PCNS‐KOH) was used as a cathode to devise a SIC. The energy storage mechanism of the cathode is shown in **Figure** **8**a where the high surface area cathode leads to EDLC wherein the anions absorbed/desorbed above 2.7 V while the cations below 2.7 V. Energy dispersive x‐ray spectroscopy (EDX) elemental mapping of cathode depicting the presence of N and P are also visible in Figure 8b. The galvanostatic charge‐discharge (GCD) profile of SIC at various current densities is shown in Figure 8c, where the device delivered reasonable energy densities of 135.3/39.6 Wh kg^−1^ at corresponding power densities of 30.0 W kg^−1^/16.1k W kg^−1^. The role of FBPCs in improving the capacitive performance was explained due to the abundant micropores, and doping of P within the carbon matrix‐induced defects which results in enlargement of carbon layers and thereby increased charge storage capacity.

##### FBPC Based Anode Material for SICs

Tamarind pod‐derived hard carbon (T‐HC) as the anode and nitrogen/sulfur co‐doped graphene (NS‐RGO) as cathode were utilized in SIC within a potential window of 1 to 4.2 V versus Na/Na^+^.^[^ ^107^ ^]^ The CV curve of SIC showed tiny humps due to the sulfur/nitrogen doping on graphene during the reduction reaction (Figure 8d). As per the electrochemical impedance spectroscopy (EIS) analysis, the material exhibited lower charge transfer resistance which suggests better conductivity of ions at the electrode/electrolyte interface. The charge storage by the adsorption and intercalation mechanism in the symmetric SIC device promotes wide charge‐discharge duration and enhanced capacitance. The adsorption/ intercalation type charge storage mechanism in the FBPC anode exhibited a long discharge time contributing to the SIC device capacitance.

In a fully carbon‐based SIC configuration, carbon nanosheets derived from pine cone shells (PSCS‐600) formed the anode, and carbon derived from a covalent triazine framework (OPDN‐CTF‐A) was utilized as the cathode.^[^ ^109^ ^]^ The charge storage mechanism is depicted in Figure 8f. The SIC device demonstrated an energy density of 111 Wh kg^−1^ at a power density of 86 W kg^−1^ and a high‐power density of 14.2k W kg^−1^ at an energy density of 40 Wh kg^−1^. In this work, dual carbon‐based SIC was developed where the role of FBPC as a battery‐type anode is that the 2D FBPC possess enough carbon interlayers for the storage of Na^+^ ions. Chemically modified carbon is also a good choice as an anodic material for SICs. Yeast‐derived carbon loaded with Nb_2_O_5_ quantum dots worked perfectly as an anodic material along with activated carbon as a cathode to deliver an energy density of 62.5 Wh kg^−1^.^[^ ^103^ ^]^ The carbon matrix helped in reducing the volume strain experienced by Nb_2_O_5_, thus imparting stability to the active material structure during the charge storage. CV curves exhibited quasi‐rectangular shapes at various scan rates, which is attributed to fast Na^+^ ion transport and a partial pseudocapacitive reaction.

Nitrogen‐doped carbon tubes (N‐MJ) decorated on their surface with graphene‐like nanosheets were prepared from metaplexis japonica fluff was used as an anode for SIC.^[^ ^110^ ^]^ The nitrogen doping (11.08%), etching, and striping using urea increased the number of active sites and surface area of the developed materials which showed a high reversible capacity (390.9 mAh g^−1^ at 50 mA g^−1^) and improved rate capability (188.1 mAh g^−1^ at 1 A g^−1^). To demonstrate the application performance in reality, the full carbon‐based SIC was assembled with the anode N‐MJ and commercial AC as a cathode. The fabricated SIC delivered an energy density of 111.4 Wh kg^−1^ at 445.8 W kg^−1^ and a power density of 2455.2 W kg^−1^ at 34.1 Wh kg^−1^ with extraordinary stability even after 5000 cycles.

##### FBPC Based Cathode Materials for SICs

With Na_2_Ti_3_O_7_ nanobelts/carbon nanofibers (CNFs) composite employed as the anode and porous carbon/CNFs as the cathode electrode in SIC, a high energy density of 126 Wh kg^−1^ and power density of 38k W kg^−1^ was obtained (Figure 8e).^[^ ^108^ ^]^ Another instance reported red willow bark‐derived porous carbon as a cathode alongside reduced graphene oxide‐supported cobalt sulphide (CoS_2_‐rGO) nanoparticles as an anode in SIC with an energy density of 112.6 Wh kg^−1^ and power density of 2.6k W kg^−1^.^[^ ^111^ ^]^ The attributes of this cathode material, include a wide potential window (ranging from 1.5 to 4.5 V Vs Na/Na^+^), a microporous structure, and EDLC behavior, collectively contributing to heightened energy density, enhanced rate capability and improved cycle life. Using rGO‐supported mixed metal sulfides is another way of enhancing the energy density of SIC.^[^ ^112^ ^]^ In the case of mixed metal sulfides, a higher energy density of 145.6 Wh kg^−1^ was achieved. The mixed metal system exhibited improved ionic and electronic conductivity that helped to achieve a high current density. In a related study, a SIC was devised by substituting CoS_2_ with NiS_2_ and employing porous carbon derived from Sichuan peppercorn as the cathode material.^[^ ^113^ ^]^ The EDLC behavior observed from the electrochemical measurements evolved from the FBPC electrode which was attributed to the presence of oxygen functional groups. The higher specific capacity achieved with the FBPC (66.7 mAh g^−1^ at 1 A g^−1^ over 100 cycles) translated to high energy densities (163.18/78.24 Wh kg^−1^) and power densities (224.99/4499.42 W kg^−1^) for the SIC device.

#### 3.1.3 Potassium Ion Capacitors (KICs)

The abundant availability of potassium (2.09 wt%) and the similar redox potential of K^+^ ions to that of Li^+^ ions make a compelling case for utilizing K^+^ in the energy storage sector for KIC. However, KICs suffer from low power density and poor cyclic stability.^[^ ^114^ ^]^ In addition, the larger ionic radii (1.38 Å) and diverse kinetics have restricted its utilization in electrodes made up of carbon‐based materials.^[^ ^115^ ^]^ Additionally, the volume of K^+^ is higher when compared to Li^+^ ion and therefore the volume will fluctuate greatly when the ions are incorporated in the electrode resulting in poor cyclic performance.^[^ ^116^ ^]^ FBPCs again are a suitable platform to test the electrochemical behavior of the KICs and some of the recent reports will be highlighted here.^[^ ^117^ ^]^

##### FBPC Based Dual Carbon Electrodes for KICs

**Figure 9:**
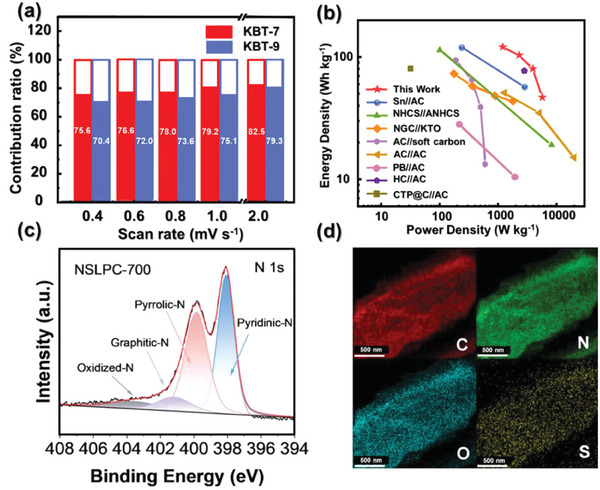
a) Adsorption and insertion contribution ratio versus scan rate for KBT‐7 and KBT‐9 anodes, b) Ragone plot comparing various KICs. Reproduced with permission.^[^ ^120^ ^]^ Copyright Elsevier 2021. c) N 1s XPS spectra, d) EDX elemental mapping of NSLPC‐700 FBPC based KIC. Reproduced with permission.^[^ ^121^ ^]^ Copyright Springer 2023.

The use of FBPCs for KICs is rather less explored, some of which are highlighted in this section. For instance, berry fruit‐derived carbon with a unique structure composed of planar surface and vertical projections (plain hill model) is a good ploy to fabricate KIC with impressive energy (178.4/115 Wh kg^−1^) and power density values (1115/10 500 W kg^−1^).^[^ ^118^ ^]^ The plain and the hill areas in the carbon structure have a heterogeneous distribution of oxygen‐containing defects which is helpful to obtain pseudocapacitive properties without compromising the conductivity of the material. These carbon structures show immense promise as electrodes. Heteroatom co‐doping in FBPCs is another facile ploy to enhance their ion transfer, conductivity, K^+^ ion storage and rate performance.^[^ ^119^ ^]^ FBPC derived from spent black tea (KBT) containing inherent N and P elements and activated carbon was used as the anode and cathode for KIC.^[^ ^120^ ^]^ **Figure** **9**a shows the pseudocapacitive contribution of KBT synthesized at two different temperatures delivering high capacitance. Ragone plot demonstrated a balanced energy and power density of this device (121.09 Wh kg^−1^ /1178.18 W kg^−1^, and 47.16 Wh kg^−1^/5658.91 W kg^−1^) compared with various other KIC devices as shown in Figure 9b.

##### FBPC Based Anode Materials for KICs

**Figure 10:**
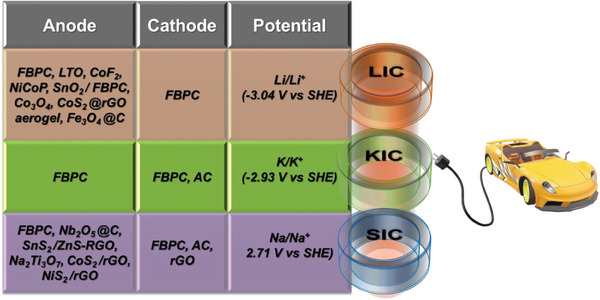
Schematic diagram showing various electrodes used for monovalent HICs recently.

In KICs, the charge storage mechanism of carbon‐based anode closely resembles that of SICs.^[^ ^122^ ^]^ This mechanism relies on adsorption and desorption, as well as K^+^ intercalation and deintercalation. Thus, the fabrication of carbon‐based anode with a hierarchical porosity and abundant functional groups/heteroatom doping finds widespread device application. Cocoon silk was utilized as a platform for the synthesis of hierarchically porous nitrogen‐doped carbon as an anode material for KIC application.^[^ ^123^ ^]^ The 3D hierarchical porous network of FBPC anode contributes to structural stability, easier electrolytic ion transportation and volume expansion buffering. The KIC device delivered an energy density of 135 Wh kg^−1^ and a power density of 112.6 W kg^−1^. A novel approach for heteroatom doping involving supermolecule mediation to create N/S co‐doped lignin‐derived FBPC (NSLPC‐700) was reported recently.^[^ ^121^ ^]^ The synthesis resulted in the formation of C_3_N_4_ as an intermediate which further decomposed within the carbon matrix to induce nitrogen doping as well as resulted in nanosheet morphology with mesoporosity. The high‐power density of 12.8k W kg^−1^ with a high energy density of 71 Wh kg^−1^ was attributed to the nitrogen‐rich functional groups confirmed by the N1s XPS spectra (Figure 9c) and the EDX mapping (Figure 9d). All these factors favored an easy diffusion of K^+^ ions. The electrode demonstrated K^+^ adsorption which was driven by diffusion rather than capacitive effects. The vacancy defects in the carbon matrix are cited as a crucial factor for the K^+^ storage. The device exhibited a high energy density of 71 Wh kg^−1^ and a high‐power density of 12,875 W kg^−1^. There are no reports of FBPC materials being utilized as cathode materials for KICs. Illustration depicting various anodes and cathodes along with the metal/metal ion potential for monovalent HICs is depicted below (**Figure** **10**).

### 3.2 Multivalent HICs

#### 3.2.1 Zinc Ion Capacitors (ZICs)

**Figure 11:**
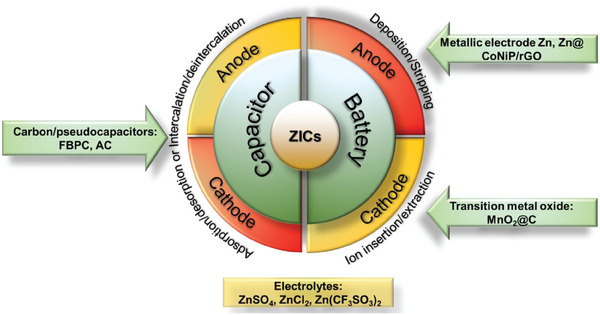
Schematic representation of various characteristics of the ZICs.

Multivalent ions‐based HICs have also evolved alongside monovalent HICs as the former can offer higher volumetric energy density than the later.^[^ ^124^ ^]^ The monovalent HICs involve highly reactive elements, which has led to the growth of multivalent HICs but the lower oxidation‐reduction potential of the multivalent metal is the major problem.^[^ ^125^ ^]^ Among various multivalent ions, ZICs are the most extensively studied ones due to the abundant availability of Zn, better safety, reduced air sensitivity, portability and the low redox potential (−0.76 V) associated with Zn^2+^ ions, and a high theoretical capacity of 823 mAh g^−1^.^[^ ^126^ ^]^ In ZIC electrode configuration, the anode and cathode are employed as battery and capacitor‐type electrodes where the charge storage mechanism may be determined by the materials. A schematic diagram is depicted in **Figure** **11** defining the two configurations of ZICs classified as i) capacitor type cathode and battery type anode ii) battery type cathode and capacitor type anode. The corresponding charge storage mechanism and materials explored are shown in the diagram along with various electrolytes used.

Commercial zinc foil is a preferred anodic material for ZICs, however, it does have limitations including non‐uniform deposition of Zn^2+^ and dendrite formation over time. Additionally, short circuit issues and safety problems are some concerns that affect the research on ZICs. Therefore, ongoing research focuses on finding suitable alternatives to Zn foil as anodic materials for ZICs.^[^ ^127^ ^]^ FBPCs are again a promising alternative for ZICs based electrodes as well and some recent instances will be highlighted in this section. A flexible ZIC device fabricated with manganese dioxide at carbon cloth composite (MnO_2_@CC) as a cathode and FBPC derived from biomass loofah (AC@CC) as an anode was reported.^[^ ^83^ ^]^ Authors suggested that during the charging/ discharging process, the evolution of byproducts such as Zn_4_(OH)_6_ SO_4_.nH_2_O on the surface of the FBPC electrode leads to an increase in the overall capacity of the device. The SEM images taken after charging the AC electrode showed the formation of intermediate flakes arising due to Zn_4_(OH)_6_ SO_4_.nH_2_O on the surface of the electrode. The multiplicative performance of the FBPC electrode in ZnSO_4_‐based electrolyte may also arise because of the capacitance retention at high current density. The authors further went on to design a flexible ZIC device with a solid electrolyte of ZnCl_2_+MnSO_4_/PVA which exhibited high energy density and power density of 676.3 µWh cm^−2^ and 2000 µW cm^−2^ respectively.

**Figure 12:**
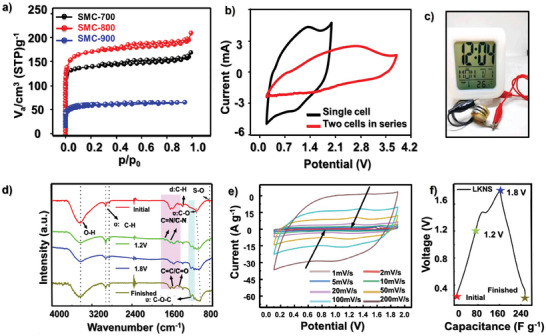
a) N_2_ adsorption‐desorption isotherm of SMC‐700, SMC‐800 and SMC‐900, b) Comparison of CV curves of single cell and two cells in series connection, c) Clock powered using three cells using SMC‐800. Reproduced with permission.^[^ ^130^ ^]^ Copyright Elsevier 2024. d) Ex situ FTIR spectra at specific charge states, e) CV curves at various scan rates and f) GCD curve of LKNS electrode. Reproduced with permission.^[^ ^131^ ^]^ Copyright American Chemical Society 2023.

There are not many reports on employing FBPC‐based anode for ZIC device fabrication because of the kinetic mismatch between the anode and cathode electrode. The sluggish intercalation‐based charge storage mechanism observed in the case of zinc ion batteries (ZIBs) and zinc‐based capacitors paved the way for substituting the transition metal oxide‐based cathode with FBPCs as seen in ZIC.^[^ ^128^, ^129^ ^]^ Thus, we present various FBPC‐based cathode materials for ZIC application in this section. With the goal of finding an efficient FBPC‐based cathode for the ZIC device, Hu et al. developed FBPC with a high surface area of 3424 m^2^ g^−1^ and a high nitrogen content from sweet potato powder.^[^ ^72a^ ^]^ The device delivered an average energy density and power density of 112 Wh kg^−1^ and 385 W Kg^−1^ respectively. Compared to this, a cathode made of FBPC derived from walnut shell along with double transition metal composite (CoNiP/rGO) exhibited a high electrochemical performance with energy densities and power densities of 143.14 /48.79 Wh kg^−1^ and 425/17k W kg^−1^ respectively. This shows the effect of incorporating the metal composites that play a significant role in enhancing the overall energy and power density of the ZIC device.^[^ ^127^ ^]^ In a recent report, Yoon and coworkers fabricated ZIC using a self‐doped heteroatoms‐rich FBPC electrode (SMC) derived from *solanum melongena*, which exhibited much exciting energy and power densities of 141.36 Wh kg^−1^ and 6935.38 W kg^−1^.^[^ ^130^ ^]^ The microporous‐rich SMCs synthesized at various temperatures led to surface areas in the range of 686.29‐ 239.16 m^2^ g^−1^ as shown in **Figure** **12**a along with CV curves of fabricated ZIC device connected in series, which can illuminate a clock (3 V) (Figure 12b,c). N/S co‐doped activated carbon derived from lignin biomass (LKNS) as cathode demonstrated an extremely high‐power density which is due to the less redox but high capacitive contribution by the FBPC electrode coupled in a ZIC device.^[^ ^131^ ^]^ Various oxygen‐rich functional groups such as hydroxyl, carboxyl and carbonyl groups and the synergetic effect of multiple heteroatoms doping provided the pseudo‐capacitive behavior (Figure 12d). FBPC electrode delivered a specific capacitance of around 307 F g^−1^ (Figure 12e,f). The device exhibited energy densities of 108.8/65.8 Wh kg^−1^ at power densities of 2880/115.2k W kg^−1^. The rice husk‐derived carbon in the ZIC attained a power density of 10k W kg^−1^ but the maximum energy density is still low (58.6 Wh kg^−1^).^[^ ^132^ ^]^ Compared to previous reports, ZIC fabricated with bamboo‐derived porous carbon‐based cathode and Zn foil anode delivered excellent energy density and power density in aqueous‐based electrolyte (166.4 Wh kg^−1^)/ 15.678k W kg^−1^) and in solid state electrolyte (197.7 Wh kg^−1^/15.22k W kg^−1^).^[^ ^133^ ^]^ Furthermore, the FBPC still retained high capacity even in a wide range of temperatures with low charge transfer resistance and high ionic conductivity. The main advantage of using lignin as the carbon source for the cathode fabrication of ZIC is that it contains inherent oxygen species which helps in preventing the desorption of electrolytic anions, resulting in enhanced self‐discharging.^[^ ^134^ ^]^

**Figure 13:**
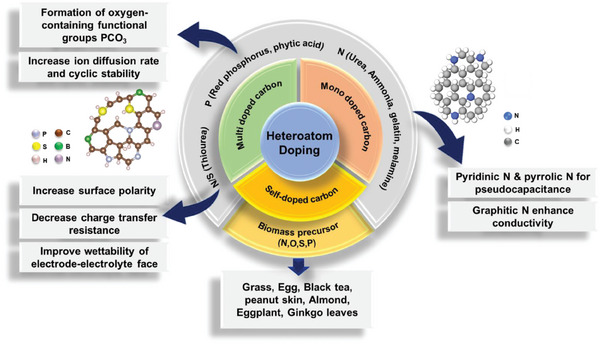
Schematic diagram showing various heteroatom doping and its advantages in enhancing the electrochemical performance of FBPCs.

Heteroatom doping is known to influence and improve the electrochemical performance of carbon‐based electrode materials, primarily by augmenting interlayer spacing, introducing defect sites, and enhancing conductivity. Ongoing research is focused on gaining a deeper understanding of the underlying mechanism of doping. Consequently, this technique finds widespread application in energy storage devices.^[^ ^135^ ^]^ **Figure** **13** depicts various heteroatom doping and its advantages in enhancing the electrochemical performance.

For example, sweet messes from glutinous rice alcoholic fermentation were used as the carbon source for the preparation of diverse and micropore‐rich FBPC.^[^ ^136^ ^]^ The oxygen‐rich functional groups present on the FBPC electrode facilitate oxidation and thereby electrostatically attract the Zn^2+^ ions in the electrolyte. The capacity of the device was mainly contributed by the FBPC which delivered an energy and power density of 116 Wh kg^−1^ and 800 W kg^−1^, respectively. An N, O co‐doped cheap wood‐derived porous carbon with uniform 2D sheet structure and a very high specific surface area (1248 m^2^ g^−1^), not only possesses a short electric/ionic transfer path, optimized wettability and conductivity for high power output but also provides a large number of interfacial active sites and improved ion adsorption capacity for charge storage. The ZIC based on these N, O co‐doped 2D carbon nanosheets exhibited a superior specific capacity of 111.0 mAh g^−1^ at 0.1 A g^−1^, high‐rate capability of 57.6%, capacity retention at a 30‐fold higher current, and a desirable energy density of 109.5 Wh kg^−1^ at 225 W kg^−1^. The ultralong cycling life it exhibits, with excellent capacity occupied after long‐duration charge and discharge cycles, is even more remarkable. These outstanding results demonstrated the viability of heteroatom doping on carbon matrix as a source for high‐performance ZIC fabrication.^[^ ^137^ ^]^ Likewise in another work, porous carbon derived from inexpensive p‐doped waste biomass was used as a cathode along with zinc foil anode for making high‐performance ZIC. The device also exhibited remarkable anti‐self‐discharge properties, maintaining 77.98% of its specific capacity observing a 72‐hour natural self‐discharge test, and long‐term cycle stability, retaining 88.2% of its initial specific capacity after 15000 cycles at 7.5 A g^−1^. Phosphorous atoms were doped into porous carbon to produce more topological imperfections and edges, which accelerated the migration of metal ions and produced outstanding energy storage performance.^[^ ^138^ ^]^

The dependence of capacitance to porosity, carbonization temperature and functional groups for developing a ZIC derived from orange peel‐based FBPC were also investigated.^[^ ^139^ ^]^ As seen in most FBPC‐based cathode electrodes, the presence of heteroatoms led to the pseudocapacitance resulting in a high‐power density of 7570 W kg^−1^ but the energy density was slightly low at 104.9 Wh kg^−1^. The electrochemical performance of cotton pulp‐derived FBPC cathode‐based ZIC was tested using a cotton paper‐derived hydrogel electrolyte with a high amount of ZnCl_2_.^[^ ^140^ ^]^ Compared to other FBPC‐based ZIC reported previously, the device exhibited a remarkable increase in energy density of 243 Wh kg^−1^ at a power density of 492 W kg^−1^ and attained a maximum power density of 8.167k W kg^−1^ because of the relatively wide potential window and high capacity of the device.

To create electronic devices that are highly flexible and durable for practical applications, researchers are continuing their investigations into the utilization of flexible gel‐based electrolytes utilizing the FBPC cathode. There are reports on the fabrication of ZIC using FBPCs but limited literature is available on developing flexible type ZICs.^[^ ^141^, ^142^ ^]^ In one such study, oil palm wood‐derived porous carbon was employed as the cathode with the hydrogel‐based electrolyte for the ZIC device, which delivered an energy density of 53.7 Wh kg^−1^ and a power density of 152.9 W kg^−1^.^[^ ^143^ ^]^ Interestingly, FBPC derived from walnut shells, doped with phosphorus, was assessed for ZIC device performance where it delivered energy densities of 56.08/127.1 Wh kg^−1^ at high power densities of 16k/400 W kg^−1^ respectively.^[^ ^138^ ^]^ The enhancement in FBPCs electrochemical performance such as surface wettability, charge transfer and diffusion rate may be explained due to the formation of PCO_3_ upon phosphorus doping which results in the development of oxygen functional groups within the porous carbon matrix. Compared to the above results, FBPC derived from aerial roots of Ficus macrocarpa was used as a cathode for ZIC where the electrochemical performance arises mostly due to the influence of pyrrolic N and carboxyl groups within the FBPC.^[^ ^144^ ^]^ Interestingly, the device exhibited a high energy density of 181.6 Wh kg^−1^. Theoretical calculations also demonstrated the influence of surface functional groups on Zn^+^ ion adsorption contributing to the overall electrochemical performance. More interestingly, a solar‐charging self‐powered flexible unit with a printed ZIC was developed using biomass kelp‐derived carbon.^[^ ^145^ ^]^ The quasi‐solid state ZIC setup exhibited an aerial energy density of 8.2 µWh cm^−2^ at a power density of 40 µW cm^−2^. Apart from the pore characteristics, the 3D structure, interconnected cell walls and the internal specific shaped channels of the FBPC results in fast ion and electron transfer simultaneously. Inspired from the high Zn^+^ storage capability of FBPC, flexible ZIC developed with agriculture waste‐derived mesoporous carbon as the cathode (2760 m^2^ g^−1^) delivered a high energy density of 32.6 µW cm^−2^ and even under the flexible condition the device still provided 11 µW cm^−2^ at a high power density of 1.906 W cm^−2^.^[^ ^59^ ^]^ Therefore, the potential window of the ZIC may be affected by the electrode, its modification, and the electrolyte which in turn affect the overall energy density and power density of the material. The electrochemical performance parameters for various FBPC based HICs explored are tabulated in **Table** **3**.

**Table 3:** Electrochemical performance of various FBPC‐based HIC electrodes.

Material (Cathode// Anode)	HIC configuration	Electrolyte	Specific capacitance / Specific capacity @ current density	Cyclic stability	Energy density [Wh kg^−1^]	Power density [W kg^−1^]	Reference
NS‐ACSC// NS‐CSC	LIC	1 M LiPF_6_	46.3 F g^−1^ at 0.05 A g^−1^	81% after 7000 cycles	101.7	113	[96]
GLPC‐900// GLPC‐600	LIC	1 M LiPF_6_	355 mAh g^−1^ at 1 A g^−1^ (half‐cell)	83% after 6000 cycles @ 2A g^−1^	118	31.6k	[102]
CSBC‐5// CoF_2_	LIC	1 M LiPF_6_	41.89 F g^−1^ at 0.05 A g^−1^	65% after 5000 cycles	82 50	190 3800	[101]
WPBC‐6// NiCoP	LIC	LiPF_6_	63.5 F g^−1^ at 0.1 A g^−1^	76.4% after 7000 cycles	127.4	18 240	[98]
HMMC// MCP‐LTO	LIC	1 M LiClO_4_/ propylene carbonate	52.3 F g^−1^	85.7% capacity retention after 10000 cycles	142 52.9	253 4556	[67]
KHPC‐K// KHPC‐600	LIC	1 M LiPF_6_	81.9 F g^−1^ at 0.1 A g^−1^	77.7% retention after 5000 cycles	169	97	[86]
JFAC// Co_3_O_4_ NS	LIC	1 M LiPF_6_	140 mAh g^−1^ (half‐cell)	87% after 3000 cycles	118	–	[100]
Porous carbon// CoS_2_@rGO aerogel	LIC	1 M LiPF_6_	114.2 mAh g^−1^ at 0.1 A g^−1^ (half‐cell)	81.5% retention after 10000 cycles	132.9 50	265 26.5k	[85]
AC// Fe_3_O_4_@C	LIC	1 M LiPF_6_	918 mAh g^−1^ at 100 mA g^−1^	83.3% after 6000 cycles	140.6 52.8	200 10k	[93]
LPC//LGC	LIC	1 M LiPF_6_	58 F g^−1^ at 0.05 A g^−1^	92.3% retention after 5000 cycles	97	11.4k	[146]
HNBC// HNBC	LIC	1 M LiPF_6_	64.2 F g^−1^ at 0.1 A g^−1^	81.9% after 10000 cycles	186.31	225	[95]
CACF// CACF	KIC	1 M KFSI	147.2 mAh g^−1^ at 10 A g^−1^	75.2% capacity retention after 10000 cycles	178.4 115	1115 10500	[118]
KBTAC// KBT	KIC	0.8 M KPF_6_	88.7mAh g^−1^ at 0.8 A g^−1^	100% columbic efficiency	121.09 47.16	1178.18 5658.91	[120]
AC// SHNPC	KIC	5 M KFSI	343 mAh g^−1^ at 50 mA g^−1^ (half‐cell)	75.4% after 3750 cycles	45	1951.8	[123]
AC//NCHC	KIC	1 M KPF_6_	42 mAh g^−1^ at 100 mA g^−1^	–	127.36	2371	[116]
AC// NSLPC	KIC	3 M KFSI	39 mAh g^−1^ at 0.05 A g^−1^	91% retention after 2000 cycles	71	92	[121]
APSRC// APSRC	SIC	1 M NaPF_6_	108.4 mAh g^−1^ at 1 A g^−1^ (half‐cell)	80.7% retention after 1000 cycles	76.4	150	[90a]
OPDN‐CTF‐A// PSCS	SIC	1 M NaClO_4_	372 mAh g^−1^ (half‐cell)	90.7% retention after 10000 cycles	111 40	86 14200	[109]
AC//T‐Nb_2_O_5_@YC	SIC	–	195.7 mAh g^−1^ at 0.05 A g^−1^ (half‐cell)	92% capacity after 2000 cycles	62.5	1250	[103]
PCPSK// SnS_2_/ZnS‐RGO	SIC	1 M NaClO_4_	148 mAh g^−1^ at 0.4 A g^−1^ (half‐cell)	100% after 1000 cycles	145.6 92.92	2250 13500	[112]
AC//N‐MJ	SIC	1 M NaClO_4_	–	100% retention after 5000 cycles	111.4 34.1	445.8 2455.2	[110]
Biomass carbon/CNF //Na_2_Ti_3_O_7_ nanobelt/ CNF	SIC	1 M NaClO_4_	111 mAh g^−1^ at 0.1 A g^−1^	90% after 1000 cycles	126 84	236 38k	[108]
RWBC// CoS_2_/rGO	SIC	1 M NaClO_4_	40.4 F g^−1^ at 0.1 A g^−1^	100% after 1000 cycles	112.6	225	[111]
SPC// NiS_2_/rGO	SIC	NaClO_4_	94.3 mAh g^−1^ at 0.3 A g^−1^ (half‐cell)	100% after 1000 cycles	163.18 78.24	224.99 4499.42	[113]
MnO_2_@CC//AC	ZIC	ZnCl_2_/MnSO4/PVA	1217.4 mF cm^−2^ at 1 mA cm^−2^	100% after 5000 cycles	676.3 µ Wh cm^−2^	2000 µW cm^−2^	[83]
BC‐CNa// Zn	ZIC	1 M ZnSO_4_	51.4 mAh g^−1^	96% after 90000 cycles	48.3	–	[141]
PC// Zn@CoNiP/rGO	ZIC	2 M ZnSO_4_	356.6 at 0.5 A g^−1^	92.2% retention after 10000 cycles at 7.5 A g^−1^	143.14 48.79	425 17k	[127]
N/S‐AC//Zn	ZIC	2 M ZnSO_4_	307 at 0.5 A g^−1^	–	65.8	115 200	[131]
ZnLFK‐PC//Zn	ZIC	2 M ZnSO_4_	123.7 mAh g^−1^	92.4% retention after 4000 cycles at 10 A g^−1^	97.8	–	[134]
RHC//Zn	ZIC	3 M Zn (CF_3_SO_3_)_2_	149.8 at 0.2 A g^−1^	95.8% after 3000 cycles at 2 A g^−1^	58.6 9.7	167.8 10k	[147]
WC//Zn	ZIC	2 M ZnSO_4_	111 mAh g^−1^ at 0.1 A g^−1^	92.7% after 50000 cycles	109.5	225	[137]
Porous carbon//Zn Flexible LSC@PI	ZIC	1 M ZnSO_4_	128.7 mF cm^−2^ at 100 mA cm^−2^	–	11 µ	1906µ	[59]
WAPC//Zn	ZIC	2 M ZnSO_4_	158.9 mAh g^−1^	88.2% after 15000 cycles	127.1	160 000	[138]
FHPCNS//Zn	ZIC	2 M ZnSO_4_	–	–	181.6 97.5	165 1.6k	[144]
PBC‐A//Zn	ZIC	1 M Zn (CF_3_SO_3_)_2_	321.3 F g^−1^ at 1 A g^−1^	78% after 20000 cycles	114.2	800	[142]
Porous carbon //Zn	ZIC	1 M ZnSO_4_	320 F g^−1^	–	111.67 41.46	158.45 12756	[148]
CDC//Zn	ZIC	ZnCl_2_ ZnCl_2_‐ cellulose hydrogel	357 F g^−1^ at 0.5 A g^−1^ 247 mAh g^−1^	85% after 20000 cycles	243	492	[140]
GRPC//Zn	ZIC	2 M ZnSO_4_	177 F g^−1^ at 0.5 A g^−1^	100% columbic efficiency	116 29	800 8000	[136]
Biomass kelp carbon//Zn	ZIC	1 M Zn (CF_3_SO_3_)_2_	445 F g^−1^ at 0.1 A g^−1^	89% after 4000 cycles	111.5	1300	[145]
LHPC//Zn	ZIC	1 M ZnSO_4_	135 F g^−1^ at 10 mV s^−1^ (half‐cell)	99% coulombic efficiency after 5800 cycles	–	–	[132]
BSCs//Zn	Solid state ZIC ZIC	PVA/KOH/Zn 1 M ZnSO_4_	124 mAh g^−1^ at 0.1 A g^−1^ 104 mAh g^−1^	94.73% after 5000 cycles 106.1% after 10000 cycles	197.7 166.4	15 221.2 15 678.8	[133]
OLDC//Zn	ZIC	2 M ZnSO_4_	306.8 mAh g^−1^ at 0.1 A g^−1^	91% after 20000 cycles	136.3 58.3	100 20k	[149]

Note: Candle soot carbon (CSC); Activated/co‐doped CSC (ACSC); Ginkgo leaf derived porous carbon (GLPC); Coconut shell biomass carbon (CSBC); Watermelon peel biomass derived carbon (WPBC); Hierarchical micro/meso porous carbons (HMMC); Mg^2+^ and Cr^3+^ co‐doped and phosphidated LTO (MCP‐ LTO); Hierarchical porous carbon (KHPC); Jack Fruit Activated Carbon (JFAC); Activated carbon (AC); Lignin derived hierarchical porous carbon (LPC); Lignin derived graphitic carbon (LGC); Hierarchically porous Nitrogen doped biomass derived carbon (HNBC); Pectin derived carbon with CaCl_2_ (CACF); Activated carbon from biomorphic carbon from kitchen bio waste (KBTAC); Cocoon silk derived hierarchically porous nitrogen doped carbon (SHPNC); Nitrogen rich biomass carbon (NCHC); Nitrogen/sulfur co‐doped lignin derived porous carbon (NSLPC); KOH activated potato starch derived carbon (APSRC); Biowaste pine cone shell derived carbon nanosheet (PSCS); Covalent triazine framework derived carbon (OPDN‐CTF‐A); Nb_2_O_5_ quantum dots confined in multichamber yeast carbon (T‐Nb_2_O_5_@YC); Peanut shell derived activated carbon (PCPSK); Nitrogen doped carbon tubes derived from metaplexis japonica fluff (N‐MJ); Carbon nanofiber (CNF); Red Willow bark derived carbon (RWBC); Sichuan peppercorn derived porous carbon (SPC); Carbon cloth (CC); Bamboo derived porous carbon (BC‐CNa); Porous carbon (PC); Zinc coordinated lignin derived porous carbon (ZnLFK‐PC); Rice husk derived carbon (RHC); Wood carbon (WC); Polyimide derived patterned laser scribed carbon (LSC@PI); Walnut shell derived carbon (WAPC); Porous carbon nanosheets functionalized with oxygen and nitrogen dopants from aerial roots of Ficus macrocarpa (FHPCNS); Porous biomass carbon (PBC); Cellulose derived carbon (CDC); Glutinous rice derived porous carbon (GRPC); Lignin derived hierarchical porous carbon (LHPC); Bamboo shavings derived porous carbon (BSC); Oxygen rich hierarchical porous carbon derived from olive leaves (OLDC).

**Figure 14:**
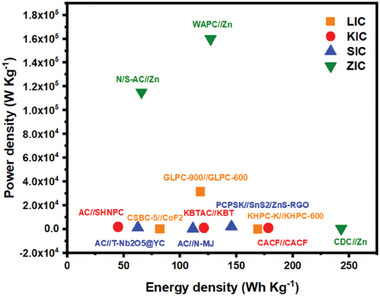
Ragone plot highlighting the variation in the energy and power densities of mono and multivalent HICs obtained with FBPCs electrodes derived from different biomass precursors (the full abbreviations of the names of precursors are provided in the footnotes of Table 3).

A closer look at Table 3 reveals that various types of FBPC materials can deliver promising results for energy and power densities for various monovalent and ZICs among multivalent HICs. In each category of HICs, it is clear that achieving the right balance between energy and power density is critical for any type of FBPC material. For the sake of comparison, we will highlight a few examples (plotted in Ragone plot **Figure** **14**) from Table 3 to illustrate which type of FBPCs can provide either high energy or power density or a balance between both values in monovalent to multivalent HICs.

For instance, in LICs, the material GLPC‐900//GLPC‐600,^[^ ^102^ ^]^ as a dual carbon electrode FBPC synthesized from Ginkgo leaves can deliver a high‐power density of 31 600 W kg^−1^, whereas KHPC‐K//KHPC‐600,^[^ ^86^ ^]^ another dual carbon FBPC electrode with a hierarchical structure can provide a high energy density of 169 Wh kg^−1^. The electrode performance in the former case was attributed to the intrinsic heteroatom doping of FBPC with O, N and S, whereas microstructure and the surface chemical properties of FBPCs played a major role in the latter case. For a multivalent ZIC, FBPC called WAPC//Zn^[^ ^138^ ^]^ derived from walnut shell delivered a high power density of 160 000 W kg^−1^ whereas another FBPC material N/S‐AC//Zn^[^ ^140^ ^]^ derived from different biomass of cotton pulp paper yielded a high energy density of 243 Wh kg^−1^. In this case, the performance of materials was either linked to phosphooxy functional groups, microstructure, and enhanced redox activity in WAPC//Zn and to abundant mesoporous and macroporous structure in N/S‐AC//Zn. These instances illustrate that it is highly unlikely to streamline the nature and properties of the FBPC and more so no single factor can serve as a performance metric for their performance for either mono or multivalent HICs.

#### 3.2.2 Other Multivalent HICs

Although FBPCs have shown great promise in monovalent HICs, their expansion across multivalent HICs, except ZICs, is limited. MICs face the predominant issue of the formation of a passivation coating on the surface of magnesium anode which limits its practical development and utilization.^[^ ^150^ ^]^ The development of CICs using FBPC as cathode or anode is still facing challenges due to the lack of suitable electrolytes and more so because it is difficult to make the room temperature energy storage device based on Ca‐based electrodes in organic solutions.^[^ ^151^ ^]^ Due to the formation of passivation coating upon the oxidation of Ca ions, the reversibility of Ca anodes is limited. On the other hand, AICs also face difficulty in their charge storage kinetics and reversibility due to the formation of a high electrostatic field around Al^3+^ ions. The large size of the Al restricts its application as an electrode material.

## 4 Critical Factors Affecting the Performance of FBPCs for HICs

As mentioned in the previous sections, FBPCs are well suited for their use either as battery‐type or supercapacitor‐type electrodes which is largely made possible due to their porous structure, surface functionalization and good conductivity. While looking at the overall success of the HICs, several factors serve as their performance index and most of these criteria rely on the nature of the electrode material chosen for the fabrication of the device.

### 4.1 Long‐Term Capacity/Cycling Stability

Any energy storage system including HIC is expected to last for a longer period to obtain the maximum output and value.^[^ ^152^ ^]^ The nature of electrode material is the primary factor that influences the quality of HICs. The higher stability of the battery and supercapacitor electrodes over a longer number of charge‐discharge cycles is crucial to achieving a long‐term capacity. The electrode materials based on FBPCs possess a great deal of stability due to the presence of hard carbon and at the same time are less prone to degradation in the longer run. The next factor controlling the long‐term stability is the nature of the electrolyte. A stable electrolyte composition is desired for the quick transport of ions over longer periods. The design of the HIC which includes the arrangement of electrode electrolyte interfaces, cell packaging, etc. can also minimize the degradation and the mechanical stress, thereby increasing the longevity. Cycling rate is another crucial factor that can impact the long‐term capacity as some HICs may perform better under a certain set of cycling rates. Lastly, the operating conditions including temperature and voltage can also determine the life of the HIC.

### 4.2 Electrode Stability

Electrode stability in HICs is primarily controlled by the type of material used for its fabrication and its utilization end; battery or supercapacitor. For instance, the battery‐type electrode material should be chosen in such a way that it can withstand repeated insertion and deinsertion with minimal degradation of the electrode, whereas the electrode material for the supercapacitor electrode should deliver high capacitance over several cycles. The stability of the electrode is also measured by the physico‐chemical properties of the material chosen, e.g., porosity, functionalization, thermal and chemical stability, surface charge etc.^[^ ^153^ ^]^

### 4.3 Electrolyte

The choice and type of electrolyte are crucial to maintaining a proper ion transport channeling between the battery anode type and supercapacitor cathode type electrodes.^[^ ^150^ ^]^ An ideal amount of ion transport leads to a good current flow which allows HIC to store and release energy. Furthermore, electrolytes can help achieve a high‐power density in the supercapacitor part of HIC through increased adsorption and desorption of the ions at the electrode/electrolyte surface. While choosing the electrolyte, it must be made sure that it is compatible with both the electrodes of HIC and does not cause degradation.^[^ ^12^ ^]^ Further, viscosity and conductivity are two factors that can influence ion mobility and transport and hence should be carefully balanced to obtain maximum performance.

### 4.4 Cell Design

The design of the cell which includes the arrangement of different components inside one compartment is also critical for achieving better and long‐lasting performance in HICs. First, the arrangement of electrodes is the main factor followed by the placement of the separator and electrolyte. The assembly has to be made in such a way as to maximize the ion transport while minimizing the electrolyte leakage or its non‐efficient distribution. The cell design has to be structurally sound so that mechanical stress and component degradation can be avoided. Overall, cell construction should be cost‐effective and efficient.^[^ ^154^ ^]^

The above factors and others including charge‐discharge ability and cycling ability are being continuously researched to better the performance of HICs. The overall efficiency including the power and energy densities and cycling stability of HICs could be enhanced by bringing innovation in electrode materials structure, the nature of electrolytes and the design of cells that can deliver optimal performance.

## 5 Conclusion and Future Perspectives

**Figure 15:**
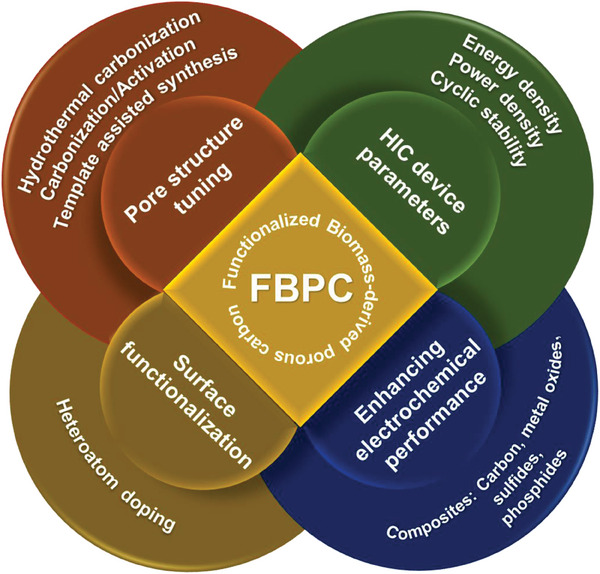
Schematic highlighting the theme of the review.

Functionalized biomass‐derived porous carbons (FBPCs) have displayed remarkable potential for hybrid ion capacitors ranging from monovalent LICs, SICs, KICs and multivalent ZICs. Several factors contribute to enhancing the electrochemical behavior of FBPCs in HICs which include but are not limited to surface area, pore type (micro or meso), surface functional groups containing heteroatoms such as O, S, N etc., and type of activation (physical or chemical) etc. This review explores recent advancements in FBPCs in terms of their synthesis and various physico‐chemical properties and their utilization as electrode materials in HICs. From the materials point of view, FBPCs are low‐cost yet effective electrode materials that have proven their worth in both batteries and supercapacitors. Their highlights such as high surface area, wide pore size distribution in both micro and meso domains, large pore volume, and high thermal and chemical stability are some of the intriguing features that are useful for electrochemical purposes. In addition, the easy manipulation of their surface with heteroatoms is another ploy to increase their productivity as electrode materials. FBPCs also present a good case where their morphology and porosity can be suitably modified using various strategies. These are also interesting materials to design composites and hybrids with other materials to suit any particular electrochemical purpose. As electrode materials for HICs, FBPCs have been used for almost all variants including LICs, SICs, KICs, and various multivalent HICs. FBPCs have been shown to deliver high capacitance, cycling stability and energy density. A plethora of literature compilation in the field necessitates a comprehensive review to provide insights into the recent highlights of the field and directions for future research. The overall theme of this review is shown in **Figure** **15**. Creating various HICs based on FBPC comes with both advantages and limitations. The detailed conclusive summary and the challenges in each section of the review are provided below.

1. The synthesis methods encompass conventional hydrothermal carbonization, pyrolysis, and template‐assisted synthesis approaches to fabricate functionalized porous carbon from biomass. Leveraging carbon derived from biomass presents both advantages and disadvantages. Among the advantages are low cost, straightforward synthesis, abundant precursor availability, moderate capacitance, cyclic stability, moderate wettability, and electrical conductivity. Conversely, the disadvantages encompass micropore‐dominated porosity, resulting in lower energy and power density. Synthesis procedures can be further improved through advanced technologies which could then bring in more optimization into the structure of the FBPCs.
2. In the design considerations for FBPCs, there is a discourse on the significance of the 2D morphology in carbon architecture as opposed to 3D carbons. Researchers in recent years have extensively examined the significance of morphological tuning in improving electrochemical performance. It has been noted that 2D porous carbons exhibit elevated capacitance compared to their 3D counterparts in ZICs. The 2D porous structure facilitates the convenient access of electrolytic ions and concurrently exhibits low charge transfer resistance, thereby enhancing capacitance. The active functional groups embedded in the carbon matrix enhance both EDLC and pseudocapacitance of the electrode material. The application of straightforward and scalable activation and functionalization methods yields FBPCs with enhanced characteristics. Further development into synthesizing FBPCs with desired dimensionalities could be beneficial for HICs. For example, exfoliation of FBPCs might be useful for battery‐type electrodes.
3. High porosity in FBPCs, indicating a substantial surface area, enhances the adsorption/desorption of ions at the electrode‐electrolyte interface and facilitates the easy diffusion of ions. The electrochemical factors dependent on porosity are detailed in the review (refer to Table 2 and Figure 5). More standardization of porosity versus performance is required. Furthermore, the limited availability of storage sites and slow diffusion kinetics in FBPC‐based energy storage devices have led to the integration of composites with high‐capacity materials such as graphene oxide, metal oxides, metal phosphides, metal sulfides, and metal carbides.
4. Introducing heteroatom doping into the carbon matrix introduces a variety of functional groups. Nitrogen functional groups, for instance, imbue the material with basic characteristics, enhancing interactions between the carbon and surface molecules. This leads to improved electrolyte‐electrode interface wettability, increased surface polarity, reduced charge transfer resistance, enhanced conductivity, accelerated ion diffusion rates, improved cyclic stability, and the formation of additional micropores for heightened electrochemical performance.
5. In contrast to SICs and LICs, which typically involve intercalation/deintercalation‐type charge storage mechanisms in anodes, the ZICs operate through the plating/stripping of Zn^2+^. Unlike cathodes based on oxides that exhibit slow kinetics, a porous carbon cathode in ZIC facilitates non‐faradaic EDLC charge storage without undergoing any phase transitions. In various HICs, the electrode configuration is vital for elucidating the charge storage mechanism. The initial setup comprises a capacitor‐type cathode and a battery‐type anode, where the former will be critical. Conversely, the alternate configuration showcases a capacitor‐based anode and a battery‐based cathode, where the latter assumes a pivotal role in influencing the overall performance of the device.
6. The complete exploration of the electrochemical charge storage mechanism in multivalent HIC‐based electrodes remains incomplete. A significant observation from the studies is the absence of research on the energy storage mechanism of multivalent HIC‐based systems, other than ZIC, after the year 2021. Variations in ionic radii, binding energy between ions and the active material, and reactivity exist between univalent and multivalent ions. These distinctions could potentially yield even more intriguing and intricate kinetics within battery‐based electrodes.

It is essential to investigate the charge storage mechanism of both monovalent and multivalent metal ions utilizing porous carbon derived from biomass. This exploration aims to achieve high energy density and power density. The emphasis can be placed on exploring diverse composite combinations with porous carbon to ensure compatibility with metal‐based anodes. Further exploration of FBPCs holds the potential for environmentally friendly contributions to the future.

## Conflict of Interest

The authors declare no conflict of interest.
